# Impact of mental disorders on the risk of heart failure among Korean patients with diabetes: a cohort study

**DOI:** 10.1186/s12933-023-01809-4

**Published:** 2023-05-18

**Authors:** Tae Kyung Yoo, Kyung-Do Han, Eun-Jung Rhee, Won-Young Lee

**Affiliations:** 1 Department of Medicine, MetroWest Medical Center, Framingham, MA USA; 2 Department of Statistics and Actuarial Science, Soongsil University, Seoul, Korea; 3 Division of Endocrinology and Metabolism, Department of Internal Medicine, Kangbuk Samsung Hospital, Sungkyunkwan University School of Medicine, Seoul, Korea

**Keywords:** Mental Disorder, Diabetes, Heart failure

## Abstract

**Background:**

Few studies have assessed the correlation between coexisting mental disorders in participants with diabetes mellitus (DM) and the risk of heart failure (HF). Herein, we conducted a cohort study to determine the association between the accumulation of mental disorders in participants with DM and the risk of HF.

**Methods:**

The Korean National Health Insurance Service records were assessed. 2,447,386 adults with DM who underwent health screening between 2009 and 2012 were analyzed. Participants with major depressive disorder, bipolar disorder, schizophrenia, insomnia, or anxiety disorders were included. In addition, participants were categorized based on the number of coexisting mental disorders. Each participant was followed until December 2018 or until the onset of HF. Cox proportional hazard modelling with confounding factors adjustment was conducted. In addition, a competing risk analysis was conducted. Subgroup analysis assessed the impact of clinical variables on the association between the accumulation of mental disorders and the risk of HF.

**Results:**

The median follow-up duration was 7.09 years. The accumulation of mental disorders was associated with a risk of HF (zero mental disorder (0), reference; 1 mental disorder, adjusted hazard ratio (aHR): 1.222, 95% confidence intervals (CI): 1.207–1.237; 2 mental disorders, aHR: 1.426, CI: 1.403–1.448; ≥3 mental disorders, aHR: 1.667, CI: 1.632–1.70. In the subgroup analysis, the strength of association was the strongest in the younger age group (< 40 years, 1 mental disorder, aHR 1.301, CI 1.143–1.481; ≥2 mental disorders, aHR 2.683, CI 2.257–3.190; 40–64 years, 1 mental disorder, aHR 1.289, CI 1.265–1.314; ≥2 mental disorders, aHR 1.762, CI 1.724–1.801; ≥65 years, 1 mental disorder, aHR 1.164, CI 1.145–1.183; ≥2 mental disorders, aHR 1.353, CI 1.330–1.377; P_inter_<0.001). In addition, income, BMI, hypertension, chronic kidney disease, history of cardiovascular disease, insulin use, and duration of DM showed significant interactions.

**Conclusions:**

Comorbid mental disorders in participants with DM are associated with an increased risk of HF. In addition, the association was stronger in a younger age group. Participants with DM and mental disorders should be monitored with increased frequency for signs of HF; for which they have a higher risk than the general population.

**Supplementary Information:**

The online version contains supplementary material available at 10.1186/s12933-023-01809-4.

## Background

Mental disorders are the leading cause of the global disease burden [1]. Previous studies have reported a prevalence of 322 million participants with depression, 264 million with anxiety disorders, 46 million with bipolar disorders, and 20 million with schizophrenia worldwide [2, 3]. Additionally, substantial evidence indicates that mental disorders are associated with other medical conditions [4]. These medical conditions are often left unattended and lead to a decreased quality of life, increased healthcare utilization, and premature death in participants with mental disorders [4, 5]. Due to the high prevalence and its impact on physical health, research is recommended to establish causal pathways between mental disorders and fatal health outcomes [1].

Previous research has shown a higher incidence of major depressive disorders, bipolar disorders, schizophrenia, anxiety disorders, and sleep disorders in participants with DM than in the general population [6]. In addition, coexisting mental disorders in participants with DM often lead to decreased adherence to treatment and an increased risk of severe complications, including blindness, amputations, stroke, and cognitive decline [7].

Another medical condition that might be affected by mental disorders includes circulatory disorders such as heart failure (HF), ischemic heart disease, peripheral artery occlusive disease, atrial fibrillation, hypertension, dyslipidemia, and stroke [4]. Momen et al. suggested that the absolute risk of developing circulatory disorders within 15 years of the onset of mental disorders is 54.1% [4]. Additionally, a recent study has suggested that severe mental disorders affect the prognosis of heart failure (HF) patients [8].

DM is known to increase the risk of HF [9, 10]; however, few large-scale studies have assessed the association between the coexisting mental health disorder in participants with DM on the risk of HF [11]. Assessing the risk of HF in DM patients with mental disorders can help us predict the prognosis of this subset of patients. Therefore, we conducted a cohort study using large datasets from the South Korean adult population to determine whether the accumulation of mental disorders in DM participants was associated with an increased risk of HF.

## Methods

### The national health insurance service records and the korean national health screening database

The National Health Insurance Service (NHIS) records and the Korean National Health Screening (KNHS) database were assessed in our study. The NHIS is a large-scale South Korean cohort [12, 13]. Approximately 50 million (97.2%) South Koreans are registered in the NHIS [14]. In South Korea, adults over 20 must undergo regular health check-ups provided by the NHIS every one to two years. Additionally, most South Koreans receive medical treatment at least once a year, with an average of 16.6 visits per person per year [14, 15]. All data and results, including patient demographics, examination findings, treatment administered to the participants, and International Classification of Diseases (ICD-10) codes from this health check-up and clinic visit, are sent to the NHIS [16]. This database forms the KNHS database, which provides information of the health-screening questionnaires and laboratory findings [12]. The Institutional Review Board (IRB) of the NHIS and Soongsil University approved this study (SSU-202,003-HR-201-01). The requirement for informed consent was waived because de-identified data were used in this study.

### Study design

**Fig. 1:**
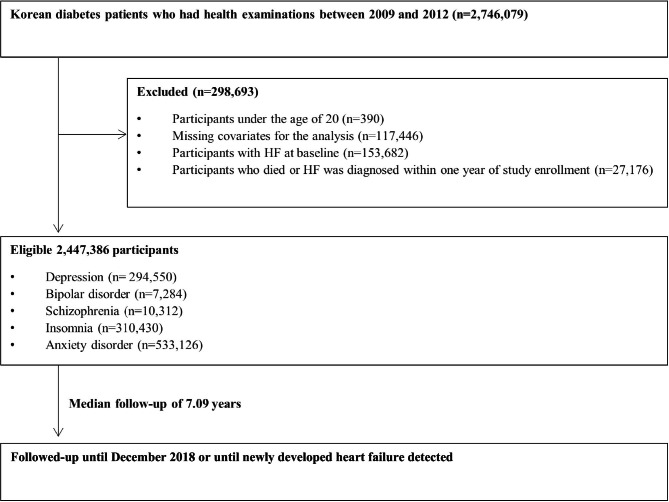
Flow diagram of study participants. Abbreviation: HF, heart failure

Data from the NHIS and KNHS databases of the South Korean population with DM who underwent health check-ups between 2009 and 2012 were assessed (n = 2,746,079). Participants with the following characteristics were excluded from the analysis: (1) age < 20 years (n = 390), (2) missing covariates for the analysis (n = 117,446), (3) participants with HF at baseline (n = 153,682), (4) participants who died within one year of enrollment in the study, and (5) those who were diagnosed with HF within one year of enrollment in the study (n = 27,176). After exclusion, 2,447,386 participants were included in this study (Fig. 1). The primary outcome was the risk of new-onset HF. The participants were followed up until December 31, 2018, or they developed newly diagnosed HF or until they died. The development of HF was assessed using the NHIS claims records at the end of 2018.

### Data collection

A standardized questionnaire was used to assess the health-related behaviors of the participants, including smoking, alcohol consumption, physical activity level, and income level [13]. Smoking status was categorized as (1) “never smoked,” (2) “ex-smokers,” or (3) “current smoker [17].” Alcohol consumption habits were categorized as (1) “no drinking,” (2) “mild drinking” (< 30 g/day), and (3) “heavy drinking” (≥ 30 g/day) [18, 19]. Regular exercise was defined as (1) participants engaged in > 25 min of high-intensity physical activity ≥ 3 days per week or (2) > 30 min of moderate-intensity physical activity ≥ 5 days per week [20]. The low-income group was defined as the total income of participants belonging to (1) the 25th percentile of the South Korean population or (2) if a patient received support for medical costs from the South Korean government.

Trained medical staff collected anthropometric data, including body weight (kg) and height (cm), using an electronic scale. Body mass index (BMI) was calculated as weight (kg) divided by height (m) squared. The participants were asked to rest for at least five minutes before the blood pressure measurement in a sitting position using a sphygmomanometer with the brachial artery at the heart level. Waist circumference (cm) was measured as the distance between the midpoint of the rib cage and the iliac crest [13]. The participants were asked to fast for at least eight hours before blood sample collection.

### Definition of medical conditions

DM was diagnosed when participants had one of the following findings at baseline: (1) a record of fasting blood glucose ≥ 126 mg/dL in the KNHS database, (2) a claim history for ICD-10 code E11–14, and (3) a record of antidiabetic medication prescription before January 2009 [21, 22]. The duration of DM and the number of oral hypoglycemic agents used were assessed by examining the claim history for ICD-10 codes E11–14 or antidiabetic medication prior to 2009. The risk of HF was assessed based on the number of baseline mental disorders. HF at baseline was defined as the presence of an ICD record (I50) and admission record due to HF prior to inclusion in the study. Incident HF was diagnosed when participants had a new ICD record of I50 and an admission record for HF during the follow-up period.

Chronic kidney disease (CKD) was defined as an estimated glomerular filtration rate of < 60 mL/min/1.73m^2^ by the Modification of Diet in the Renal Disease method [23]. Hypertension was defined when participants had one of the following conditions: (1) a systolic blood pressure ≥ 140 mmHg, (2) a diastolic blood pressure ≥ 90 mmHg, (3) the presence of ICD codes ICD 10–15, and (4) an antihypertensive medication claim history. Dyslipidemia was defined when participants had one of the following criteria: (1) a total cholesterol level ≥ 240 mg/dL or (2) an ICD code E78 with dyslipidemia medication claim history [13].

The participants were divided into four groups (0, 1, 2, ≥ 3 mental disorders) based on the number of coexisting mental disorders at baseline using ICD-10 codes: major depressive disorder (F32, F33), bipolar disorder (F30, F31), schizophrenia (F20), insomnia (F47.0, F51.0), and anxiety disorder (F40, F41). Participants with a diagnosis code within five years of study inclusion were considered to have mental disorders at baseline. The participants with multiple mental disorders were counted separately. For example, participants with depression and bipolar disorder were counted once in the depression category and once in the bipolar disorder category and were categorized into the two mental disorders group.

### Statistical methods

Data were expressed as mean ± standard deviation or geometric mean (95% CI) based on the distribution of continuous variables. Categorical variables were expressed as frequencies (%). The One-way Analysis of Variance (ANOVA) was used to compare the means of continuous variables, while the chi-square test was used to compare the means of categorical variables. Incidence was calculated per 1,000 person-years.

The Cox proportional hazards model was used to assess the association between the number of coexisting mental disorders and the risk of HF. In addition, the association between each mental disorder and the risk of HF was assessed. Hazard ratios (HRs) and 95% CIs were calculated. Five different sequential models were created for the multivariable analysis. Model 1 was a crude model. Model 2 was minimally adjusted for biological factors (age and sex). Model 3 was adjusted for lifestyle and social factors, in addition to Model 2 (age, sex, BMI, low income, smoking, drinking, and regular exercise). Model 4 was adjusted for past medical history, DM treatment, and severity, in addition to Model 3 (age, sex, BMI, low income, smoking, drinking, regular exercise, hypertension, dyslipidemia, CKD, fasting glucose, duration of DM, insulin use, duration of DM ≥ 5 years, and use of more than three types of oral hypoglycemic agents). Finally, Model 5 was adjusted for CVD at baseline in addition to Model 4. As mortality events could compete with the outcome, a competing risk analysis was conducted using the Fine and Gray model by adjusting for confounding factors for all models. The assumption of proportionality was verified using Schoenfeld residuals and log-log plots. Harrell’s C-index and Akaike Information Criterion (AIC) were calculated in the multivariable models to compare the predictive power of survival models. The original model assessed the predictive power of models 1–5. The mental disorder accumulation model was created to compare whether adding a mental disorder accumulation variable to the original model strengthens the predictive power. In addition, Integrated Discrimination Index (IDI) and Net Reclassification Index (NRI) were calculated for reclassification improvement analysis to compare the predictive power of models with and without mental disorders. The models without competing risk analysis were used for Harrell’s C-index, AIC, IDI, and NRI calculation. The cumulative incidence function curve from the full-adjusted model (Model 5) was plotted using the Kaplan-Meier method.

The participants were further divided into three subgroups: (1) 0 mental disorder, (2) 1 mental disorder, and (3) ≥ 2 mental disorders. Interaction analyses were conducted in each subgroup to determine the clinical factors (age, sex, income, BMI, smoking, drinking, regular exercise, hypertension, dyslipidemia, CKD, CVD, insulin use, number of oral hypoglycemic agents used, and duration of DM) that could affect the association between the number of mental disorders and the risk of HF. Multiplicative interaction terms were used to test the interactions. A new term created by multiplying the two variables was added to the Cox regression model. Multiple testing was adjusted by using Sidak method. In addition, participants were divided into three groups according to age (20–39 years, 40–64 years, and ≥ 65 years old group) to assess the impact of aging. Two-sided p-values of < 0.05 were defined as statistically significant. SAS (Statistical Analysis Software 9.4; SAS Institute Inc., Cary, North Carolina, USA) was used to conduct all statistical analyses.

## Results

### Baseline characteristics

The median follow-up duration was 7.09 (5.89–8.06) years. Among the participants, 1,660,732 had 0 mental disorder, 503,414 had 1 mental disorder, 201,357 had 2 mental disorders, and 81,883 had ≥ 3 mental disorders. 32.14% (n = 786,654) of patients had mental disorders. All clinical characteristics showed significant differences between the groups (Table 1). The mean age of patients with mental disorders was 61.14 ± 11.22 years, and that of participants without mental disorders was 54.84 ± 12.16 years (p < 0.001). Participants without mental disorders had a higher proportion of men (68.95%), smoking rate (30.77%), heavy drinking rate (12.01%), systolic blood pressure (129.18 ± 15.76 mmHg), fasting glucose level (148.29 ± 47.69 mg/dL), and total cholesterol level (198.21 ± 42.38 mg/dL) (p < 0.001). The mental disorder group had lower BMIs (25.11 ± 3.39 kg/m^2^ vs. 24.92 ± 3.38 kg/m^2^), systolic blood pressures (129.18 ± 15.76 mmHg vs. 128.56 ± 15.81 mmHg), waist circumferences (85.53 ± 8.6 cm vs. 84.98 ± 8.68 cm), LDL-C (111.83 ± 41.12 mg/dL vs. 110.72 ± 41.17 mg/dL), and heavy drinking rates (12.01% vs. 6.62%) than the group of participants without mental disorders (p < 0.001) (**Supplemental Table S1**). When we compared characteristics based on each mental disorder, all variables showed significant differences, except for age (p = 0.113) and eGFR (p = 0.059), between patients with and without bipolar disorder; dyslipidemias (p = 0.056), CKD (p = 0.702), CVD (p = 0.236), LDL-C level (p = 0.137), and eGFR (p = 0.355) between participants with and without schizophrenia **(Supplemental Table S2)**.

**Table 1:** Baseline characteristics according to the number of mental disorders

	Number of mental disorders				p-value	p for trend
	0	1	2	≥ 3		
n	1,660,732	503,414	201,357	81,883		
Age, years	54.84 ± 12.16	60.16 ± 11.36	62.63 ± 10.77	63.46 ± 10.6	< 0.0001	< 0.0001
Age group, years					< 0.0001	< 0.0001
< 40	172,310 (10.38)	18,404 (3.66)	3617 (1.8)	1053 (1.29)		
40–64	1,116,744 (67.24)	297,779 (59.15)	105,680 (52.48)	40,678 (49.68)		
≥ 65	371,678 (22.38)	187,231 (37.19)	92,060 (45.72)	40,152 (49.04)		
Sex					< 0.0001	< 0.0001
Men	1,145,066 (68.95)	244,885 (48.64)	78,081 (38.78)	28,114 (34.33)		
Women	515,666 (31.05)	258,529 (51.36)	123,276 (61.22)	53,769 (65.67)		
Income, Q1 (Lowest)	341,567 (20.57)	108,084 (21.47)	44,733 (22.22)	19,073 (23.29)	< 0.0001	< 0.0001
Smoking					< 0.0001	< 0.0001
Non	811,873 (48.89)	324,409 (64.44)	142,901 (70.97)	59,766 (72.99)		
Ex	337,898 (20.35)	81,607 (16.21)	27,241 (13.53)	9642 (11.78)		
Current	510,961 (30.77)	97,398 (19.35)	31,215 (15.5)	12,475 (15.24)		
Drinking					< 0.0001	< 0.0001
Non	820,369 (49.4)	333,872 (66.32)	151,442 (75.21)	65,795 (80.35)		
Mild	640,898 (38.59)	131,264 (26.07)	39,407 (19.57)	12,808 (15.64)		
Heavy	199,465 (12.01)	38,278 (7.6)	10,508 (5.22)	3280 (4.01)		
Regular exercise	349,544 (21.05)	104,194 (20.7)	40,210 (19.97)	15,632 (19.09)	< 0.0001	< 0.0001
Hypertension	861,072 (51.85)	307,321 (61.05)	131,726 (65.42)	54,076 (66.04)	< 0.0001	< 0.0001
Dyslipidemia	629,360 (37.9)	231,149 (45.92)	102,340 (50.83)	44,041 (53.79)	< 0.0001	< 0.0001
CKD	146,018 (8.79)	65,575 (13.03)	32,686 (16.23)	15,114 (18.46)	< 0.0001	< 0.0001
CVD	23,244 (1.4)	12,928 (2.57)	7307 (3.63)	3862 (4.72)	< 0.0001	< 0.0001
DM Duration, ≥ 5 years	446,980 (26.91)	179,791 (35.71)	80,347 (39.9)	33,450 (40.85)	< 0.0001	< 0.0001
Insulin	105,866 (6.37)	52,216 (10.37)	28,176 (13.99)	13,883 (16.95)	< 0.0001	< 0.0001
OHA, ≥ 3 types	212,974 (12.82)	83,483 (16.58)	36,545 (18.15)	15,057 (18.39)	< 0.0001	< 0.0001
Depression	0 (0)	96,242 (19.12)	117,533 (58.37)	80,775 (98.65)	< 0.0001	< 0.0001
Bipolar	0 (0)	1159 (0.23)	1739 (0.86)	4386 (5.36)	< 0.0001	< 0.0001
Schizophrenia	0 (0)	3394 (0.67)	2867 (1.42)	4051 (4.95)	< 0.0001	< 0.0001
Insomnia	0 (0)	117,535 (23.35)	113,063 (56.15)	79,832 (97.5)	< 0.0001	< 0.0001
Anxiety	0 (0)	285,084 (56.63)	167,512 (83.19)	80,530 (98.35)	< 0.0001	< 0.0001
BMI, kg/m^2^	25.11 ± 3.39	24.98 ± 3.36	24.85 ± 3.4	24.72 ± 3.46	< 0.0001	< 0.0001
Waist Circumference, cm	85.53 ± 8.6	85.08 ± 8.62	84.84 ± 8.74	84.67 ± 8.9	< 0.0001	< 0.0001
SBP, mmHg	129.18 ± 15.76	128.77 ± 15.78	128.47 ± 15.82	127.51 ± 15.89	< 0.0001	< 0.0001
DBP, mmHg	79.55 ± 10.32	78.41 ± 10.06	77.89 ± 10.06	77.43 ± 10.11	< 0.0001	< 0.0001
Fasting glucose, mg/dL	148.29 ± 47.69	140.58 ± 44.81	136.98 ± 44.81	134.86 ± 45.52	< 0.0001	< 0.0001
Total cholesterol, mg/dL	198.21 ± 42.38	195.19 ± 42.81	193.86 ± 43.45	193.7 ± 44.38	< 0.0001	< 0.0001
HDL-C, mg/dL	51.92 ± 22.92	52.29 ± 24.34	52.51 ± 25.16	52.52 ± 26.31	< 0.0001	< 0.0001
LDL-C, mg/dL	111.83 ± 41.12	111.02 ± 40.94	110.25 ± 41.5	110.04 ± 41.73	< 0.0001	< 0.0001
eGFR	86.75 ± 36.72	83.82 ± 35.27	81.78 ± 34.94	80.55 ± 34.54	< 0.0001	< 0.0001
TG	149.71 (149.57–149.84)	141.21 (140.99–141.42)	139.08 (138.75–139.41)	139.98 (139.47–140.5)	< 0.0001	< 0.0001

Continuous variables are expressed as mean ± standard deviation, and categorical variables are expressed as frequency (percent)

Abbreviations: Q, quartile; CKD, chronic kidney disease; CVD, cardiovascular disease; DM, diabetes mellitus; OHA, oral hypoglycemic agent; BMI, body mass index; SBP, systolic blood pressure; DBP, diastolic blood pressure; HDL-C, high-density lipoprotein cholesterol; LDL-C, low-density lipoprotein cholesterol; eGFR, estimated glomerular filtration rate; TG, triglyceride

### Risk of HF according to the number of coexisting mental disorders

**Fig. 2:**
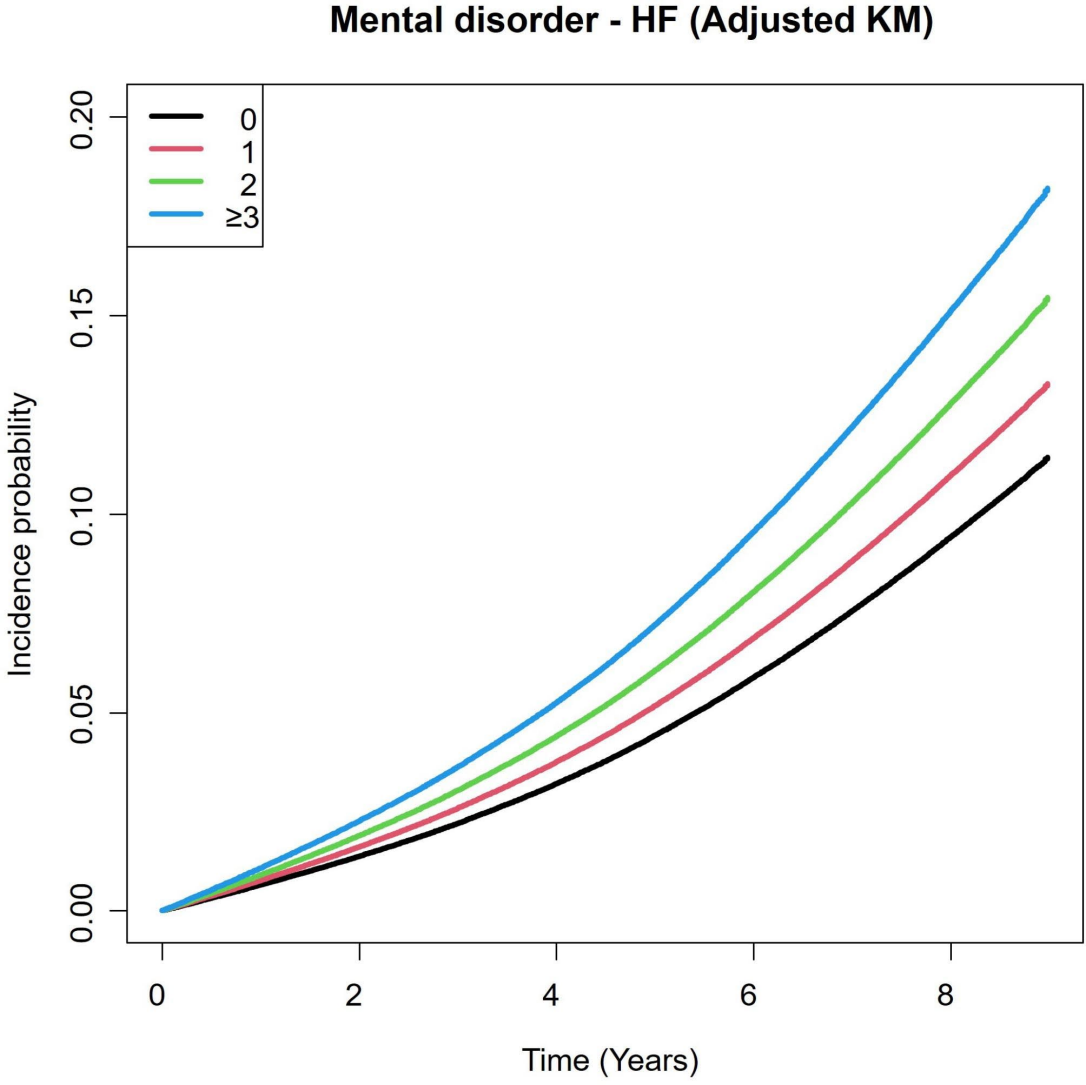
**Cumulative incidence curve using adjusted Kaplan-Meier methods** X axis = time (Years), Y axis = incidence probability. 0: 0 mental disorder; 1: 1 mental disorder; ≥ 2: ≥ 2 mental disorders.

The risk of HF in participants with mental disorders was significantly associated with the number of mental disorders in the crude model. The strength of the association showed a graded increase with an increasing number of coexisting mental disorders in the crude model (Table 2, **competing risk model 1**). The associations were significant after adjusting for confounding factors in multiple sequential models (Table 2, **Competing risk model 2–5**). The cumulative incidence curve showed increased incidence probability with a higher number of coexisting mental disorders (Fig. 2). Adding the mental disorder accumulation variable to the predictive model (mental disorder accumulation model) showed a higher c-index in all models compared to the original model. In addition, AIC values were lower in the mental disorder accumulation model (**Supplemental Table S3, S4**). IDI and NRI values were higher than 0, indicating that a model with a mental disorder accumulation predicts the risk of HF better than a model without it (**Supplemental Table S5).**

**Table 2:** Risk of heart failure according to the number of coexisting mental disorders

	N	HF	Duration	Rate	Competing risk Model 1	Competing risk Model 2	Competing risk Model 3	Competing risk Model 4	Competing risk Model 5
**Number of mental disorders**									
0	1,660,732	83,675	11,389,796.56	7.3465	1 (Ref.)	1 (Ref.)	1 (Ref.)	1 (Ref.)	1 (Ref.)
1	503,414	40,048	3,374,050.53	11.8694	1.598 (1.579, 1.617)	1.273 (1.257, 1.288)	1.269 (1.253, 1.284)	1.225 (1.211, 1.241)	1.222 (1.207, 1.237)
2	201,357	21,035	1,313,248.76	16.0175	2.141 (2.109, 2.174)	1.541 (1.517, 1.566)	1.530 (1.507, 1.555)	1.434 (1.411, 1.457)	1.426 (1.403, 1.448)
≥ 3	81,883	10,280	514,046.18	19.9982	2.667 (2.612, 2.722)	1.852 (1.813, 1.891)	1.831 (1.793, 1.870)	1.684 (1.649, 1.721)	1.667 (1.632, 1.703)

The risk of heart failure was expressed as HR with 95% confidence interval (CI).

The incidence rate was calculated per 1000 person-years

Model 1: crude model

Model 2: age, sex

Model 3: age, sex, BMI, low income, smoking, drinking, and regular exercise

Model 4: Age, sex, BMI, low income, smoking, drinking, regular exercise, hypertension, dyslipidemia, CKD, fasting glucose, duration of DM, insulin use, duration of DM ≥ 5 years, and use of more than three types of oral hypoglycemic agents

Model 5: Age, sex, BMI, low income, smoking, drinking, regular exercise, hypertension, dyslipidemia, CKD, CVD, fasting glucose, duration of DM, insulin use, duration of DM ≥ 5 years, and use of more than three types of oral hypoglycemic agents

HF, heart failure; Ref, reference; CKD, chronic kidney disease; CVD, cardiovascular disease; DM, diabetes mellitus; BMI, body mass index; HR, hazard ratio

When we assessed the **e**ach mental disorder separately, each mental disorder was associated with an increased risk of HF. This association was significant after adjusting for confounding factors (competing risk model 5: depression, HR, 1.321; CI, 1.304–1.338; bipolar disorder HR, 1.406; CI, 1.301–1.520; schizophrenia HR, 1.405; CI, 1.302–1.516; insomnia, HR, 1.305; CI, 1.289–1.322; anxiety disorder, HR, 1.287; CI, 1.273–1.302) (**Supplemental Table S6**).

### Subgroup analysis

**Fig. 3:**
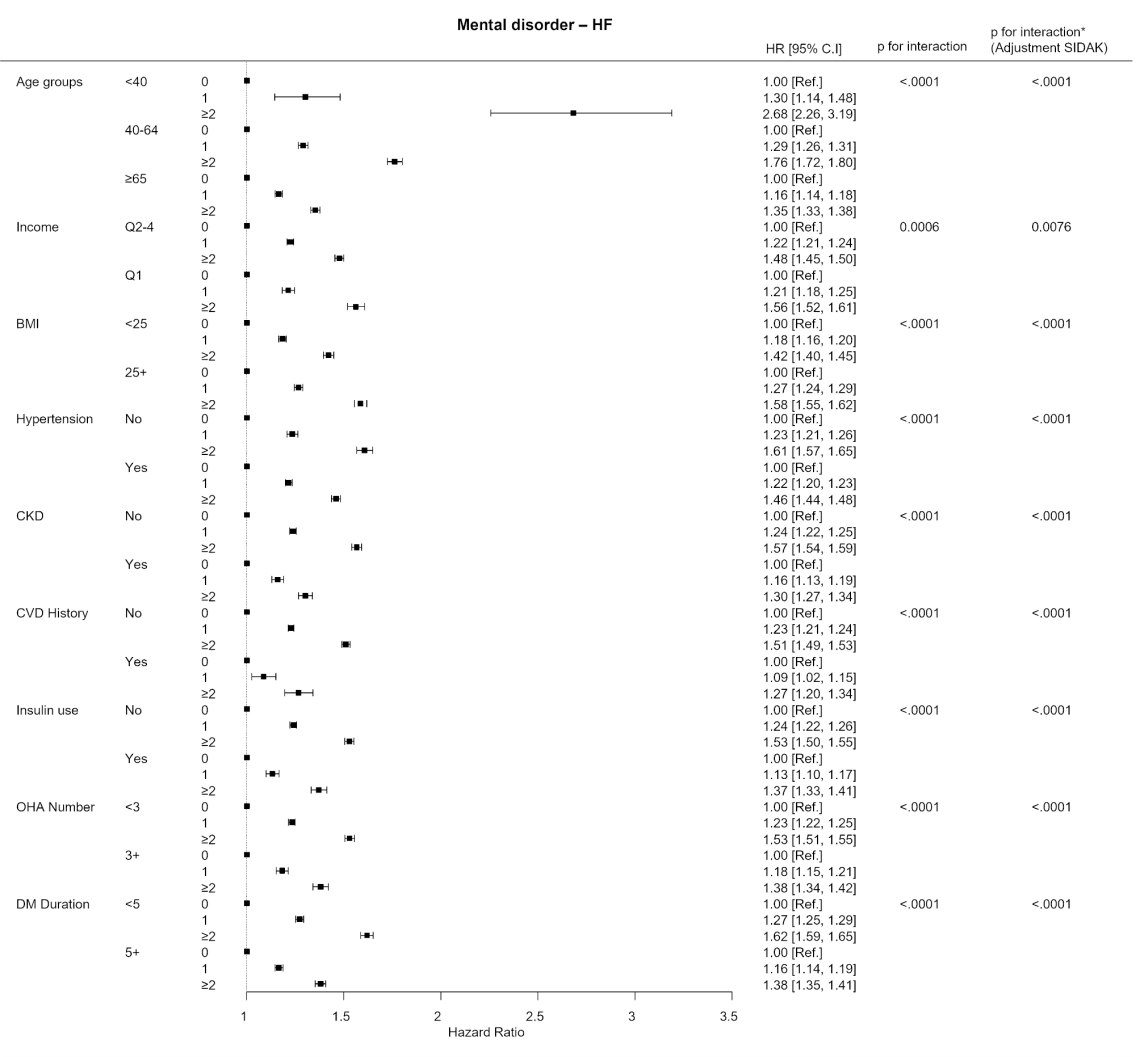
**Forest plot of subgroup analysis result** X axis = adjusted hazard ratio, Y axis = mental disorders. Abbreviations: Ref, reference; HR, hazard ratio; C.I, confidence interval; BMI, body mass index; CKD, chronic kidney disease; CVD, cardiovascular disease; OHA, oral hypoglycemic agent; DM, diabetes mellitus

Interaction analysis and subsequent subgroup analysis were performed. In the < 40 years age group, the impact of mental disorder accumulation was stronger than that in the older age groups (either 40–64 or ≥ 65 years). In addition, mental disorder accumulation had a stronger impact on the low-income group (Q1), with higher BMI (≥ 25 kg/m^2^), no-hypertension, no-CKD, no-CVD history, no-insulin use, less than three types of OHA use, and less than five years of DM group. (Fig. 3; Table 3).

**Table 3:** Subgroup analysis according to the number of mental disorders

		Number of mental disorder	N	HF	Duration	Rate	Competeing risk Model 5 (95% CI)	*P for interaction
Age groups	< 40	0	172,310	1908	1201397.99	1.5881	1 (Ref.)	**< 0.0001**
		1	18,404	259	126184.98	2.0525	1.301 (1.143, 1.481)	
		≥ 2	4670	137	31480.83	4.3519	2.683 (2.257, 3.190)	
	40–64	0	1,116,744	40,225	7762206.14	5.1822	1 (Ref.)	
		1	297,779	14,992	2050684.93	7.3107	1.289 (1.265, 1.314)	
		≥ 2	146,358	10,549	980653.12	10.7571	1.762 (1.724, 1.801)	
	≥ 65	0	371,678	41,542	2426192.43	17.1223	1 (Ref.)	
		1	187,231	24,797	1197180.62	20.7128	1.164 (1.145, 1.183)	
		≥ 2	132,212	20,629	815,161	25.3067	1.353 (1.330, 1.377)	
Sex	Male	0	1,145,066	56,389	7814725.73	7.2157	1 (Ref.)	0.416
		1	244,885	19,780	1610896.73	12.2789	1.229 (1.208, 1.249)	
		≥ 2	106,195	12,119	659098.17	18.3872	1.472 (1.442, 1.502)	
	Female	0	515,666	27,286	3575070.83	7.6323	1 (Ref.)	
		1	258,529	20,268	1763153.8	11.4953	1.216 (1.194, 1.238)	
		≥ 2	177,045	19,196	1168196.78	16.4322	1.512 (1.484, 1.541)	
Income	Q2-4	0	1,319,165	64,967	9062904.67	7.1685	1 (Ref.)	**0.0076**
		1	395,330	31,433	2659638.76	11.8185	1.224 (1.207, 1.241)	
		≥ 2	219,434	24,280	1426903.54	17.0159	1.477 (1.454, 1.500)	
	Q1	0	341,567	18,708	2326891.89	8.0399	1 (Ref.)	
		1	108,084	8615	714411.77	12.0589	1.213 (1.182, 1.245)	
		≥ 2	63,806	7035	400391.41	17.5703	1.562 (1.519, 1.607)	
BMI (kg/m^2^)	< 25	0	844,070	44,954	5750494.61	7.8174	1 (Ref.)	**< 0.0001**
		1	263,600	21,319	1746031.44	12.21	1.184 (1.164, 1.203)	
		≥ 2	153,822	16,889	975093.25	17.3204	1.421 (1.395, 1.448)	
	25+	0	816,662	38,721	5639301.94	6.8663	1 (Ref.)	
		1	239,814	18,729	1628019.09	11.5042	1.266 (1.244, 1.288)	
		≥ 2	129,418	14,426	852201.69	16.9279	1.585 (1.554, 1.617)	
Smoking	Non, ex	0	1,149,771	60,328	7921183.55	7.616	1 (Ref.)	0.4682
		1	406,016	32,725	2736769.06	11.9575	1.221 (1.205, 1.238)	
		≥ 2	239,550	26,547	1557851.58	17.0408	1.484 (1.462, 1.507)	
	Current	0	510,961	23,347	3468613.01	6.7309	1 (Ref.)	
		1	97,398	7323	637281.46	11.491	1.219 (1.187, 1.252)	
		≥ 2	43,690	4768	269443.36	17.6957	1.554 (1.505, 1.605)	
Drinking	Non, Mild	0	1,461,267	74,983	10028685.57	7.4769	1 (Ref.)	0.8101
		1	465,136	37,500	3119475.89	12.0213	1.226 (1.210, 1.241)	
		≥ 2	269,452	30,022	1740087.29	17.2532	1.504 (1.483, 1.526)	
	Heavy	0	199,465	8692	1361110.99	6.386	1 (Ref.)	
		1	38,278	2548	254574.64	10.0089	1.252 (1.198, 1.308)	
		≥ 2	13,788	1293	87207.65	14.8267	1.604 (1.512, 1.701)	
Regular exercise	No	0	1,311,188	66,811	8959839.95	7.4567	1 (Ref.)	0.8664
		1	399,220	32,667	2661619.93	12.2734	1.219 (1.202, 1.235)	
		≥ 2	227,398	25,954	1455521.57	17.8314	1.486 (1.464, 1.509)	
	Yes	0	349,544	16,864	2429956.6	6.94	1 (Ref.)	
		1	104,194	7381	712430.6	10.3603	1.233 (1.199, 1.267)	
		≥ 2	55,842	5361	371773.38	14.4201	1.539 (1.492, 1.587)	
Hypertension	No	0	799,660	26,575	5532114.8	4.8038	1 (Ref.)	**< 0.0001**
		1	196,093	10,376	1334150.19	7.7772	1.234 (1.206, 1.263)	
		≥ 2	97,438	7649	641715.4	11.9196	1.607 (1.566, 1.649)	
	Yes	0	861,072	57,100	5857681.75	9.7479	1 (Ref.)	
		1	307,321	29,672	2039900.34	14.5458	1.215 (1.198, 1.233)	
		≥ 2	185,802	23,666	1185579.54	19.9615	1.460 (1.437, 1.483)	
Dyslipidemia	No	0	1,031,372	48,200	7064268.54	6.8231	1 (Ref.)	1
		1	272,265	20,425	1816591.38	11.2436	1.222 (1.202, 1.243)	
		≥ 2	136,859	14,392	874979.27	16.4484	1.490 (1.461, 1.519)	
	Yes	0	629,360	35,475	4325528.01	8.2013	1 (Ref.)	
		1	231,149	19,623	1557459.14	12.5994	1.221 (1.200, 1.243)	
		≥ 2	146,381	16,923	952315.67	17.7704	1.501 (1.473, 1.530)	
CKD	No	0	1,514,714	67,810	10416080.13	6.5101	1 (Ref.)	**< 0.0001**
		1	437,839	30,519	2955953.15	10.3246	1.237 (1.220, 1.255)	
		≥ 2	235,440	23,102	1535964.61	15.0407	1.566 (1.542, 1.591)	
	Yes	0	146,018	15,865	973716.43	16.2932	1 (Ref.)	
		1	65,575	9529	418097.38	22.7913	1.159 (1.129, 1.189)	
		≥ 2	47,800	8213	291330.33	28.1914	1.301 (1.266, 1.337)	
CVD History	No	0	1,637,488	80,578	11241155.6	7.1681	1 (Ref.)	**< 0.0001**
		1	490,486	37,989	3294519.44	11.531	1.228 (1.213, 1.243)	
		≥ 2	272,071	29,164	1762004.36	16.5516	1.510 (1.489, 1.532)	
	Yes	0	23,244	3097	148640.96	20.8354	1 (Ref.)	
		1	12,928	2059	79531.08	25.8892	1.086 (1.025, 1.150)	
		≥ 2	11,169	2151	65290.59	32.945	1.266 (1.196, 1.340)	
Insulin use	No	0	1,554,866	71,973	10701079.56	6.7258	1 (Ref.)	**< 0.0001**
		1	451,198	32,616	3045178.98	10.7107	1.240 (1.223, 1.256)	
		≥ 2	241,181	23,783	1575053.52	15.0998	1.528 (1.504, 1.552)	
	Yes	0	105,866	11,702	688,717	16.991	1 (Ref.)	
		1	52,216	7432	328871.54	22.5985	1.132 (1.099, 1.166)	
		≥ 2	42,059	7532	252241.43	29.8603	1.371 (1.331, 1.413)	
OHA Number	< 3	0	1,447,758	67,235	9917565.36	6.7794	1 (Ref.)	**< 0.0001**
		1	419,931	31,222	2811278.27	11.106	1.232 (1.215, 1.249)	
		≥ 2	231,638	24,384	1493841.85	16.323	1.530 (1.506, 1.554)	
	3+	0	212,974	16,440	1472231.2	11.1667	1 (Ref.)	
		1	83,483	8826	562772.25	15.6831	1.182 (1.151, 1.213)	
		≥ 2	51,602	6931	333453.09	20.7855	1.380 (1.341, 1.420)	
DM Duration	< 5	0	1,213,752	47,295	8332764.95	5.6758	1 (Ref.)	**< 0.0001**
		1	323,623	20,470	2176231.39	9.4062	1.272 (1.251, 1.293)	
		≥ 2	169,443	15,412	1100868.29	13.9999	1.619 (1.588, 1.650)	
	5+	0	446,980	36,380	3057031.61	11.9004	1 (Ref.)	
		1	179,791	19,578	1197819.13	16.3447	1.165 (1.145, 1.186)	
		≥ 2	113,797	15,903	726426.66	21.8921	1.380 (1.353, 1.407)	

Competing risk Model 5: Adjusted for age, sex, BMI, low income, smoking, drinking, regular exercise, hypertension, dyslipidemia, CKD, CVD, fasting glucose, duration of DM ≥ 5 years, insulin use, and use of more than three types of oral hypoglycemic agents

*Multiple testing was adjusted with Sidak method

Abbreviations: Q, quartile; HR, hazard ratio; CI, confidence interval; BMI, body mass index; DM, diabetes mellitus; CKD, chronic kidney disease; CVD, cardiovascular disease; OHA, oral hypoglycemic agent

The baseline characteristics of each subgroup and the follow-up duration were compared. In the age subgroups, the follow-up duration was shorter in the < 40 years group than in the ≥ 65 years age group, and the proportion of men was significantly higher in the < 40 years group (81.72%) than in the ≥ 65 years group. The mean age of the low- and high-income groups was similar (56.88 ± 11.67 vs. 56.86 ± 12.37, p = 0.247). However, compared with the CKD subgroup, the mean age of the no-CKD subgroup was significantly lower (55.89 ± 11.99 vs. 65.06 ± 11, p < 0.0001) (**Supplemental Table S7, S8**).

The subgroup analysis according to mental disorders (**Supplemental Table S9-S13**) revealed that both men and women with depression had an increased risk of HF (**Supplemental Table S9**). In addition, smoking, drinking, and regular exercise did not contribute to the development of HF in the schizophrenia group (**Supplemental Table S11**).

In addition, we conducted a subgroup analysis according to age. Participants were divided into 20–39 years, 40–64 years, and ≥ 65 years old. Variables did not show a significant interaction in the 20–39 years group. Sex, obesity, number of OHA use, and DM duration showed significant interactions in the 40–64 years group. The strength of association between mental disorder accumulation and the risk of HF was stronger in the women, obese, and less than three types of OHA use, and less than five years of DM group in 40–64 years. In ≥65 years old group, obesity, CKD, and DM duration showed significant interactions. The CKD group showed a weaker association with the risk of HF than the no-CKD group (**Supplemental Tables 14–16**).

## Discussion

This is a novel study assessing the association between mental disorders and HF in a large-scale DM population. Our results showed that an increased number of coexisting mental disorders was associated with a risk of HF in a population with DM. In addition, mental disorders such as depression, bipolar disorders, schizophrenia, insomnia, and anxiety disorders were significantly associated with the risk of HF in participants with DM. The impact of mental disorder accumulation was the strongest in the young age group (≤40). This association was consistent after adjusting for confounding factors and competing risk analysis.

DM and mental disorders have a bidirectional relationship. Individuals with DM are at an increased risk for mental disorders due to continuous distress, such as feeling overwhelmed by the DM regimen, concerns about possible complications, and guilt when their DM is not managed well [7]. Conversely, many first- and second-generation antipsychotic medications increase the risk of type 2 DM, obesity, and dyslipidemia [24]. Additionally, participants with severe mental disorders have multiple barriers to effective control of DM, such as stress, isolation, periods of deteriorating mental health, low self-efficacy, lack of social support, and poor relationships with healthcare providers [25].

Even though numerous studies demonstrate a clear relationship between mental health and CVDs, the impact of mental disorders on the development of HF has rarely been investigated [26]. Poliwartek et al. conducted a study in a US population of 20,906 participants. They showed that participants with severe mental disorders, such as schizophrenia, bipolar disorder, and severe depression, presented with clinical HF seven years earlier than the general population. Additionally, men with severe mental disorders showed higher mortality rates than those without [8]. However, this study did not investigate the risk of HF. Our study is unique as we used a larger population and specifically included participants with DM. Williams et al. prospectively assessed the risk of HF in 2,501 participants. They showed that depression was associated with a greater risk of HF in older women but not in older men. However, the study results were limited because of the small number of participants with depression (n = 188; 132 women and 56 men) [27]. In our subgroup analysis, depression in both men and women was significantly associated with an increased risk of HF. In addition, the risk of HF was consistently elevated in all age groups in the depression group. It is possible that the relatively small number of participants masked the results of the previous study.

Previously, an unhealthy lifestyle in patients with mental disorders, including increased smoking, a high-calorie diet, higher intake of cholesterol and carbohydrate-rich diet, and decreased exercise time, which leads to metabolic disturbances, was suggested as a cause of the increased risk of CVDs in these patients [26, 28–30]. Interestingly, participants with mental disorders in our cohort had lower smoking rates and BMIs than those without mental disorders. Furthermore, the elevated HF risk in the participants with mental disorders persisted after adjusting for social and lifestyle-related confounding factors such as BMI, smoking, drinking, income, and physical activity levels. These findings suggest that in addition to medications that can cause metabolic disturbances and unhealthy lifestyles in participants with mental disorders, mental disorders *per se* can directly impact HF development in participants with DM. Furthermore, our findings suggest that multiple coexisting mental disorders can increase the risk of HF in an additive fashion in participants with DM.

The impact of mental disorders on the development of HF can be explained through multiple mechanisms. First, hyperactivation of the sympathetic nervous system in participants with mental disorders may contribute to HF development. Depression, insomnia, schizophrenia, and anxiety disorders are associated with increased serum adrenaline levels [26, 31–33]. In addition, the noradrenaline metabolite is increased in the serum of participants with untreated bipolar disorder compared to that in healthy controls [34]. Additionally, antipsychotics can increase plasma catecholamine levels [35]. This leads to chronically elevated sympathetic neural signals in the heart, potentially contributing to HF development [36].

Second, altered left ventricular (LV) structure and cardiac remodeling can reduce the LV ejection fraction and lead to HF [8]. This could be due to the intrinsic effects of mental disorders, in addition to the poor lifestyle habits of participants with mental disorders and the effects of antipsychotic drug treatment [8, 37]. Pillinger et al. showed that early diffuse fibroinflammatory myocardial process is present in medicated participants with schizophrenia compared to a matched healthy cohort. The results suggested that myocardial fibrosis and inflammation in participants with schizophrenia are due to antipsychotic treatment or factors intrinsic to schizophrenia [37]. This potential intrinsic effect of schizophrenia or antipsychotic medications on the heart might explain the results of the subgroup analysis of our study; there were no interactions with behavioral factors in the schizophrenia group. Finally, the medication effect could have contributed to the increased HF risk. Tricyclic antidepressants can also decrease cardiac contractility [38]. Lithium has a potential depressant effect on the myocardium [39]. Atypical antipsychotics can indirectly contribute to the development of HF by increasing sympathetic activity, thereby causing weight gain and insulin resistance [39].

Our subgroup analysis revealed several interesting findings. First, the impact of mental disorder accumulation was stronger in the younger age groups than in the older age groups. This might be due to desensitization of the aged heart against adrenergic stimulation from mental disorders [26, 31–34, 40, 41].

Second, the low-income group was more affected by mental disorder accumulation, although there was no significant difference in age between the low- and higher-income groups. Low-income households are known to be associated with a lifetime risk of mental disorders [42]. In addition, household income is strongly associated with heart disease [43]. This finding suggests that low income might potentiate the detrimental effects of mental disorder accumulation on HF development.

Third, participants with CKD were less affected by the accumulation of mental disorders, especially in age ≥65 years. Correcting for multiple cardiovascular risk factors, such as hypertension, diabetes, and dyslipidemia does not neutralize the impact of CKD on the risk of CVD [44]. CKD may be a more potent driving factor for HF than mental disorder accumulation in participants with CKD, especially in the older adult group.

Fourth, CVD history, insulin use, the number of oral hypoglycemic agents used (OHA, ≥ 3 types), and duration of DM (≥ 5 years) all showed strong interactions. Participants with these characteristics were less affected by mental disorder accumulation, older, and more frequently associated with other CVD risk factors, such as hypertension, dyslipidemia, and CKD. The presence of multiple cardiovascular risk factors in this population may have partially masked the effects of mental disorder accumulation.

Of note, sex interaction was insignificant in the risk of HF in participants with DM and concurrent mental disorders. The impact of mental disorder accumulation on the risk of HF might be similar in both men and women.

Our study has several strengths. First, we included a large cohort with DM from the South Korean population. Second, in addition to a large number of participants, our cohort represented the community because we used an established nationwide cohort, the NHIS records [14]. Third, we extensively assessed the association between mental disorders and the risk of HF in participants with DM by conducting rigorous statistical analysis.

### Limitations

Our study had some limitations. First, it was a retrospective study; however, having a large cohort size, our study can reliably suggest a relationship between mental disorders and the risk of HF. Second, the study was conducted in a single ethnicity, South Korean. Finally, the analysis did not include participants who developed HF after 2018. To mitigate this limitation, we tracked the presence of mental disorders five years before inclusion in the study. This provided an adequate follow-up period to assess the risk of HF. Prospective studies focusing on broader ethnic groups and longer follow-up durations should be conducted to confirm our study results.

## CONCLUSIONS

In conclusion, our results show that the presence and accumulation of mental disorders in participants with DM are significantly associated with an increased risk of HF. Therefore, participants with DM and comorbid mental disorders should be monitored more frequently for HF development than the general population.

## Electronic supplementary material

Below is the link to the electronic supplementary material.

Supplementary Material 1

## Data Availability

The datasets were derived from sources in the public domain: [National Health Information Database of the National Health Insurance Service, https://nhiss.nhis.or.kr/].

## List of abbreviations

HF: Heart failure
DM: Diabetes mellitus
HR: Hazard ratio
CI: Confidence interval
CVD: Cardiovascular disease
NHIS: National health insurance service
KNHS: Korean national health screening
ICD-10: International Classification of Diseases
IRB: Institutional Review Board
BMI: Body mass index
CKD: Chronic kidney disease
SAS: Statistical Analysis System
eGFR: Estimated glomerular filtration rate
LDL-C: Low density lipoprotein cholesterol
HDL-C: High density lipoprotein cholesterol
LV: Left ventricle
Q: Quartile
OHA: Oral hypoglycemic agent
SBP: Systolic blood pressure
DBP: Diastolic blood pressure

## References

[1] Global regional. and national burden of 12 mental disorders in 204 countries and territories, 1990–2019: a systematic analysis for the Global Burden of Disease Study 2019. *Lancet Psychiatry* 2022, 9(2):137–150. 10.1016/S2215-0366(21)00395-3 PMC8776563 35026139

[2] World Health O. Depression and other common mental disorders: global health estimates. In. Geneva: World Health Organization; 2017.

[3] Global regional (2018). National incidence, prevalence, and years lived with disability for 354 diseases and injuries for 195 countries and territories, 1990–2017: a systematic analysis for the global burden of Disease Study 2017. Lancet.

[4] Momen NC, Plana-Ripoll O, Agerbo E, Benros ME, Børglum AD, Christensen MK, Dalsgaard S, Degenhardt L, de Jonge P, Debost J-CPG (2020). Association between Mental Disorders and subsequent medical conditions. N Engl J Med.

[5] Rancans E, Renemane L, Kivite-Urtane A, Ziedonis D (2020). Prevalence and associated factors of mental disorders in the nationwide primary care population in Latvia: a cross-sectional study. Ann Gen Psychiatry.

[6] Robinson DJ, Coons M, Haensel H, Vallis M, Yale JF (2018). Diabetes and Mental Health. Can J Diabetes.

[7] Ducat L, Philipson LH, Anderson BJ (2014). The mental health comorbidities of diabetes. JAMA.

[8] Polcwiartek C, Loewenstein D, Friedman DJ, Johansson KG, Graff C, Sørensen PL, Nielsen RE, Kragholm K, Torp-Pedersen C, Søgaard P (2021). Clinical heart failure among patients with and without severe Mental Illness and the Association with Long-Term Outcomes. Circulation: Heart Failure.

[9] Chung SM, Lee J-I, Han E, Seo H-A, Jeon E, Kim HS, Yoon JS (2022). Association between the diabetes drug cost and Cardiovascular events and death in Korea: a National Health Insurance Service Database Analysis. Endocrinol Metab.

[10] Yu J, Lee S-H, Kim MK (2022). Recent updates to clinical practice guidelines for diabetes Mellitus. Endocrinol Metab.

[11] Kenny HC, Abel ED (2019). Heart failure in type 2 diabetes Mellitus. Circul Res.

[12] Rhee EJ, Kwon H, Park SE, Han KD, Park YG, Kim YH, Lee WY (2020). Associations among obesity degree, Glycemic Status, and risk of Heart failure in 9,720,220 korean adults. Diabetes Metab J.

[13] Cho JH, Kwon HM, Park SE, Jung JH, Han KD, Park YG, Kim YH, Rhee EJ, Lee WY (2020). Protective effect of smoking cessation on subsequent myocardial infarction and ischemic stroke independent of weight gain: a nationwide cohort study. PLoS ONE.

[14] Understanding and Utilizing Claim Data from the Korean National Health Insurance Service(NHIS) and Health Insurance Review & Assessment (HIRA) Database for Research. *J Lipid Atheroscler* 2022, 11:0. 10.12997/jla.2022.11.2.103 PMC9133780 35656154

[15] OECD. OECD Reviews of Public Health. Korea; 2020.

[16] Song SO, Jung CH, Song YD, Park CY, Kwon HS, Cha BS, Park JY, Lee KU, Ko KS, Lee BW (2014). Background and data configuration process of a nationwide population-based study using the korean national health insurance system. Diabetes Metab J.

[17] Ryan H, Trosclair A, Gfroerer J. Adult Current Smoking: Differences in Definitions and Prevalence Estimates—NHIS and NSDUH, 2008. *Journal of Environmental and Public Health* 2012, 2012:918368. 10.1155/2012/918368 PMC3357540 22649464

[18] Traversy G, Chaput J-P (2015). Alcohol consumption and obesity: an update. Curr Obes Rep.

[19] Agarwal DP (2002). Cardioprotective effects of light–moderate consumption of alcohol: a review of putative mechanisms. Alcohol Alcohol.

[20] Lanier JB, Bury DC, Richardson SW (2016). Diet and Physical Activity for Cardiovascular Disease Prevention. Am Fam Physician.

[21] Committee ADAPP (2021). 2. Classification and diagnosis of diabetes: Standards of Medical Care in Diabetes—2022. Diabetes Care.

[22] Hur KY, Moon MK, Park JS, Kim SK, Lee SH, Yun JS, Baek JH, Noh J, Lee BW, Oh TJ (2021). 2021 clinical practice guidelines for diabetes Mellitus of the korean Diabetes Association. Diabetes Metab J.

[23] Levey AS, Bosch JP, Lewis JB, Greene T, Rogers N, Roth D (1999). A more accurate method to estimate glomerular filtration rate from serum creatinine: a new prediction equation. Modification of Diet in Renal Disease Study Group. Ann Intern Med.

[24] Mangurian C, Newcomer JW, Modlin C, Schillinger D (2016). Diabetes and Cardiovascular Care among people with severe Mental illness: a Literature Review. J Gen Intern Med.

[25] Mulligan K, McBain H, Lamontagne-Godwin F, Chapman J, Flood C, Haddad M, Jones J, Simpson A (2018). Barriers to effective diabetes management – a survey of people with severe mental illness. BMC Psychiatry.

[26] Chaddha A, Robinson EA, Kline-Rogers E, Alexandris-Souphis T, Rubenfire M (2016). Mental Health and Cardiovascular Disease. Am J Med.

[27] Williams SA, Kasl SV, Heiat A, Abramson JL, Krumholz HM, Vaccarino V (2002). Depression and risk of heart failure among the elderly: a prospective community-based study. Psychosom Med.

[28] Wrzosek M, Wojnar M, Sawicka A, Tałałaj M, Nowicka G (2018). Insomnia and depressive symptoms in relation to unhealthy eating behaviors in bariatric surgery candidates. BMC Psychiatry.

[29] Dipasquale S, Pariante CM, Dazzan P, Aguglia E, McGuire P, Mondelli V (2013). The dietary pattern of patients with schizophrenia: a systematic review. J Psychiatr Res.

[30] Lopresti AL, Jacka FN (2015). Diet and bipolar disorder: a review of its relationship and potential therapeutic mechanisms of action. J Altern Complement Med.

[31] Alvarenga ME, Richards JC, Lambert G, Esler MD (2006). Psychophysiological mechanisms in panic disorder: a correlative analysis of noradrenaline spillover, neuronal noradrenaline reuptake, power spectral analysis of heart rate variability, and psychological variables. Psychosom Med.

[32] Han KS, Kim L, Shim I (2012). Stress and sleep disorder. Exp Neurobiol.

[33] Breier A, Wolkowitz OM, Roy A, Potter WZ, Pickar D (1990). Plasma norepinephrine in chronic schizophrenia. Am J Psychiatry.

[34] Zumárraga M, Dávila R, Basterreche N, Arrue A, Goienetxea B, Zamalloa MI, Erkoreka L, Bustamante S, Inchausti L, González-Torres MA (2010). Catechol O-methyltransferase and monoamine oxidase a genotypes, and plasma catecholamine metabolites in bipolar and schizophrenic patients. Neurochem Int.

[35] Boyda HN, Ho AA, Tse L, Procyshyn RM, Yuen JWY, Kim DD, Honer WG, Barr AM. Differential Effects of Acute Treatment With Antipsychotic Drugs on Peripheral Catecholamines.Frontiers in Psychiatry2020,11. 10.3389/fpsyt.2020.617428 PMC7735989 33335492

[36] Lymperopoulos A, Rengo G, Koch WJ (2013). Adrenergic nervous system in heart failure: pathophysiology and therapy. Circul Res.

[37] Pillinger T, Osimo EF, de Marvao A, Berry MA, Whitehurst T, Statton B, Quinlan M, Brugger S, Vazir A, Cook SA (2019). Cardiac structure and function in patients with schizophrenia taking antipsychotic drugs: an MRI study. Translational Psychiatry.

[38] Zima AV, Qin J, Fill M, Blatter LA (2008). Tricyclic antidepressant amitriptyline alters sarcoplasmic reticulum calcium handling in ventricular myocytes. Am J Physiol Heart Circ Physiol.

[39] Manolis TA, Manolis AA, Manolis AS (2019). Cardiovascular Safety of Psychiatric Agents: a cautionary tale. Angiology.

[40] Leineweber K, Wangemann T, Giessler C, Bruck H, Dhein S, Kostelka M, Mohr F-W, Silber R-E, Brodde O-E (2002). Age-dependent changes of cardiac neuronal noradrenaline reuptake transporter (uptake1) in the human heart. J Am Coll Cardiol.

[41] Leenen FHH, Coletta E, Fourney A, White R (2005). Aging and cardiac responses to epinephrine in humans: role of neuronal uptake. Am J Physiol Heart Circ Physiol.

[42] Sareen J, Afifi TO, McMillan KA, Asmundson GJ (2011). Relationship between household income and mental disorders: findings from a population-based longitudinal study. Arch Gen Psychiatry.

[43] Lemstra M, Rogers M, Moraros J (2015). Income and heart disease: neglected risk factor. Can Fam Physician.

[44] Jankowski J, Floege J, Fliser D, Böhm M, Marx N (2021). Cardiovascular Disease in chronic kidney disease. Circulation.

