# Supplementary material for: Impact of mental disorders on the risk of heart failure among Korean patients with diabetes: a cohort study
Source: Cardiovasc Diabetol. 2023 May 18;22:115. doi: 10.1186/s12933-023-01809-4 (PMC10197825; doi:10.1186/s12933-023-01809-4)
Supplement: Supplementary file 1 — Supplementary Material 1 [file 12933_2023_1809_MOESM1_ESM.docx]

**Supplemental Table S1. Baseline characteristics according to the presence of mental disorder**

|  | Mental disorder | | |
| --- | --- | --- | --- |
|  | No | Yes | p-value |
| n | 1,660,732 | 786,654 |  |
| Age, years | 54.84 ± 12.16 | 61.14 ± 11.22 | <.001 |
| Age group, years |  |  | <.001 |
| <40 | 172,310 (10.38) | 23,074 (2.93) |  |
| 40-64 | 1,116,744 (67.24) | 444,137 (56.46) |  |
| ≥65 | 371,678 (22.38) | 319,443 (40.61) |  |
| Sex |  |  | <.001 |
| Men | 1,145,066 (68.95) | 351,080 (44.63) |  |
| Women | 515,666 (31.05) | 435,574 (55.37) |  |
| Income, Q1 (Lowest) | 341,567 (20.57) | 171,890 (21.85) | <.001 |
| Smoking |  |  | <.001 |
| Non | 811,873 (48.89) | 52,7076 (67) |  |
| Ex | 337,898 (20.35) | 118,490 (15.06) |  |
| Current | 510,961 (30.77) | 141,088 (17.94) |  |
| Drinking |  |  | <.001 |
| Non | 820,369 (49.4) | 551,109 (70.06) |  |
| Mild | 640,898 (38.59) | 183,479 (23.32) |  |
| Heavy | 199,465 (12.01) | 52,066 (6.62) |  |
| Regular exercise | 349,544 (21.05) | 160,036 (20.34) | <.001 |
| Hypertension | 861,072 (51.85) | 493,123 (62.69) | <.001 |
| Dyslipidemia | 629,360 (37.9) | 377,530 (47.99) | <.001 |
| CKD | 146,018 (8.79) | 113,375 (14.41) | <.001 |
| CVD | 23,244 (1.4) | 24,097 (3.06) | <.001 |
| DM Duration, ≥5 years | 446,980 (26.91) | 293,588 (37.32) | <.001 |
| Insulin | 105,866 (6.37) | 94,275 (11.98) | <.001 |
| OHA, ≥3 types | 212,974 (12.82) | 135,085 (17.17) | <.001 |
| Depression | - | 294,550 (37.44) | <.001 |
| Bipolar | - | 7,284 (0.93) | <.001 |
| Schizophrenia | - | 10,312 (1.31) | <.001 |
| Insomnia | - | 310,430 (39.46) | <.001 |
| Anxiety | - | 533,126 (67.77) | <.001 |
| BMI, kg/m^2^ | 25.11 ± 3.39 | 24.92 ± 3.38 | <.001 |
| Waist Circumference, cm | 85.53 ± 8.6 | 84.98 ± 8.68 | <.001 |
| SBP, mmHg | 129.18 ± 15.76 | 128.56 ± 15.81 | <.001 |
| DBP, mmHg | 79.55 ± 10.32 | 78.17 ± 10.07 | <.001 |
| Fasting glucose, mg/dL | 148.29 ± 47.69 | 139.07 ± 44.93 | <.001 |
| Total cholesterol, mg/dL | 198.21 ± 42.38 | 194.69 ± 43.15 | <.001 |
| HDL-C, mg/dL | 51.92 ± 22.92 | 52.37 ± 24.76 | <.001 |
| LDL-C, mg/dL | 111.83 ± 41.12 | 110.72 ± 41.17 | <.001 |
| eGFR | 86.75 ± 36.72 | 82.96 ± 35.13 | <.001 |

Continuous variables are expressed as mean ± standard deviation, and categorical variables are expressed as frequency (percent).

Abbreviations: Q, quartile; CKD, chronic kidney disease; DM, diabetes mellitus; OHA, oral hypoglycemic agent; BMI, body mass index; SBP, systolic blood pressure; DBP, diastolic blood pressure; HDL-C, high-density lipoprotein cholesterol; LDL-C, low-density lipoprotein cholesterol; eGFR, estimated glomerular filtration rate.

**Supplemental Table S2. Baseline characteristics according to mental disorders**

|  | Depression | | | Bipolar | | | Schizophrenia | | |
| --- | --- | --- | --- | --- | --- | --- | --- | --- | --- |
|  | No | Yes | p-value | No | Yes | p-value | No | Yes | p-value |
| n | 2,152,836 | 294,550 |  | 2,440,102 | 7,284 |  | 2,437,074 | 10,312 |  |
| Age group | 56.17±12.22 | 61.89±10.96 | <.001 | 56.86±12.22 | 57.09±11.65 | 0.113 | 56.88±12.23 | 53.1±10.81 | <.001 |
| Age group |  |  | <.001 |  |  | <.001 |  |  | <.001 |
| <40 | 188,556(8.76) | 6,828(2.32) |  | 195,015(7.99) | 369(5.07) |  | 194,721(7.99) | 663(6.43) |  |
| 40-64 | 1,400,144(65.04) | 160,737(54.57) |  | 1,555,978(63.77) | 4,903(67.31) |  | 1,552,865(63.72) | 8,016(77.73) |  |
| ≥65 | 564,136(26.2) | 126,985(43.11) |  | 689,109(28.24) | 2,012(27.62) |  | 689,488(28.29) | 1,633(15.84) |  |
| Sex |  |  | <.001 |  |  | <.001 |  |  | <.001 |
| Men | 1,373,879(63.82) | 122,267(41.51) |  | 1,492,614(61.17) | 3,532(48.49) |  | 1,490,899(61.18) | 5,247(50.88) |  |
| Women | 778,957(36.18) | 172,283(58.49) |  | 947,488(38.83) | 3,752(51.51) |  | 946,175(38.82) | 5,065(49.12) |  |
| Income, Q1  (Lowest) | 448,985(20.86) | 64,472(21.89) | <.001 | 511,452(20.96) | 2,005(27.53) | <.001 | 509,150(20.89) | 4,307(41.77) | <.001 |
| Smoking |  |  | <.001 |  |  | <.001 |  |  | <.001 |
| Non | 1,136,108(52.77) | 202,841(68.86) |  | 1,334,526(54.69) | 4,423(60.72) |  | 1,332,761(54.69) | 6,188(60.01) |  |
| Ex-smoker | 414,213(19.24) | 42,175(14.32) |  | 455,476(18.67) | 912(12.52) |  | 455,379(18.69) | 1,009(9.78) |  |
| Current | 602,515(27.99) | 49,534(16.82) |  | 650,100(26.64) | 1,949(26.76) |  | 648,934(26.63) | 3,115(30.21) |  |
| Drinking |  |  | <.001 |  |  | <.001 |  |  | <.001 |
| Non | 1,154,024(53.6) | 217,454(73.83) |  | 1,366,057(55.98) | 5,421(74.42) |  | 1,363,182(55.94) | 8,296(80.45) |  |
| Mild | 763,925(35.48) | 60,452(20.52) |  | 822,858(33.72) | 1,519(20.85) |  | 822,716(33.76) | 1,661(16.11) |  |
| Heavy | 234,887(10.91) | 16,644(5.65) |  | 251,187(10.29) | 344(4.72) |  | 251,176(10.31) | 355(3.44) |  |
| Regular exercise | 451,128(20.96) | 58,452(19.84) | <.001 | 508,206(20.83) | 1,374(18.86) | <.001 | 508,044(20.85) | 1,536(14.9) | <.001 |
| Hypertension | 1,165,725(54.15) | 188,470(63.99) | <.001 | 1,350,511(55.35) | 3,684(50.58) | <.001 | 1,349,821(55.39) | 4,374(42.42) | <.001 |
| Dyslipidemia | 857,139(39.81) | 149,751(50.84) | <.001 | 1,003,487(41.12) | 3,403(46.72) | <.001 | 1,002,552(41.14) | 4,338(42.07) | 0.056 |
| CKD | 211,772(9.84) | 47,621(16.17) | <.001 | 258,497(10.59) | 896(12.3) | <.001 | 258,312(10.6) | 1,081(10.48) | 0.702 |
| CVD | 36,260 (1.68) | 11,081 (3.76) | <.001 | 47,117 (1.93) | 224 (3.08) | <.001 | 47,125 (1.93) | 216 (2.09) | 0.236 |
| DM Duration,  ≥5 years | 621,483(28.87) | 119,085(40.43) | <.001 | 738,134(30.25) | 2,434(33.42) | <.001 | 737,663(30.27) | 2,905(28.17) | <.001 |
| Insulin | 156,678(7.28) | 43,463(14.76) | <.001 | 199,235(8.17) | 906(12.44) | <.001 | 199,138(8.17) | 1,003(9.73) | <.001 |
| OHA, ≥3 | 294,389(13.67) | 53,670(18.22) | <.001 | 346,809(14.21) | 1,250(17.16) | <.001 | 346,399(14.21) | 1,660(16.1) | <.001 |
| Depression | - | - | - | 290,161(11.89) | 4,389(60.26) | <.001 | 290,311(11.91) | 4,239(41.11) | <.001 |
| Bipolar | 2,895(0.13) | 4,389(1.49) | <.001 | - | - | - | 5,920(0.24) | 1,364(13.23) | <.001 |
| Schizophrenia | 6,073(0.28) | 4,239(1.44) | <.001 | 8,948(0.37) | 1,364(18.73) | <.001 | - | - | - |
| Insomnia | 200,819(9.33) | 109,611(37.21) | <.001 | 306,883(12.58) | 3,547(48.7) | <.001 | 306,836(12.59) | 3,594(34.85) | <.001 |
| Anxiety | 368,454(17.11) | 164,672(55.91) | <.001 | 529,154(21.69) | 3,972(54.53) | <.001 | 529,142(21.71) | 3,984(38.63) | <.001 |
| BMI | 25.07±3.38 | 24.85±3.42 | <.001 | 25.04±3.39 | 25.73±3.79 | <.001 | 25.04±3.38 | 25.83±4.01 | <.001 |
| Waist  Circumference | 85.41±8.61 | 84.91±8.79 | <.001 | 85.35±8.63 | 87.15±9.54 | <.001 | 85.34±8.63 | 87.41±10.04 | <.001 |
| SBP | 129.1±15.77 | 128.13±15.83 | <.001 | 128.99±15.78 | 125.2±15.27 | <.001 | 129±15.78 | 123.74±15.7 | <.001 |
| DBP | 79.29±10.27 | 77.81±10.1 | <.001 | 79.11±10.26 | 77.34±10.01 | <.001 | 79.12±10.26 | 77.17±10.42 | <.001 |
| Fasting glucose | 146.36±47.08 | 137.76±45.86 | <.001 | 145.33±47.01 | 141.99±49.65 | <.001 | 145.32±46.98 | 146.01±55.32 | 0.14 |
| Total cholesterol | 197.59±42.51 | 193.32±43.55 | <.001 | 197.08±42.66 | 195.14±44.09 | <.001 | 197.08±42.65 | 196.47±45.26 | 0.148 |
| HDL -C | 52.03±23.28 | 52.27±25.29 | <.001 | 52.07±23.54 | 50.21±19.59 | <.001 | 52.08±23.53 | 49.03±22.28 | <.001 |
| LDL -C | 111.71±41.09 | 109.79±41.5 | <.001 | 111.48±41.14 | 110.44±40.76 | 0.032 | 111.47±41.14 | 112.08±42.78 | 0.137 |
| eGFR | 86.02±36.37 | 81.96±35.21 | <.001 | 85.53±36.27 | 84.73±34.11 | 0.059 | 85.53±36.26 | 85.86±36.54 | 0.355 |

**Supplemental Table S2- continued.**

|  | Insomnia | | | Anxiety | | |
| --- | --- | --- | --- | --- | --- | --- |
|  | No | Yes | p-value | No | Yes | p-value |
|  | 2,136,956 | 310,430 |  | 1,914,260 | 533,126 |  |
| Age group | 56.04±12.17 | 62.47±11.05 | <.001 | 55.6±12.22 | 61.4±11.09 | <.001 |
| Age group |  |  | <.001 |  |  | <.001 |
| <40 | 188,695(8.83) | 6,689(2.15) |  | 180,981(9.45) | 14,403(2.7) |  |
| 40-64 | 1,398,209(65.43) | 162,672(52.4) |  | 1,263,471(66) | 297,410(55.79) |  |
| ≥65 | 550,052(25.74) | 141,069(45.44) |  | 469,808(24.54) | 221,313(41.51) |  |
| Sex |  |  | <.001 |  |  | <.001 |
| Men | 1,366,899(63.96) | 129,247(41.63) |  | 1,269,390(66.31) | 226,756(42.53) |  |
| Women | 770,057(36.04) | 181,183(58.37) |  | 644,870(33.69) | 306,370(57.47) |  |
| Income, Q1  (Lowest) | 443,526(20.76) | 69,931(22.53) | <.001 | 398,278(20.81) | 115,179(21.6) | <.001 |
| Smoking |  |  | <.001 |  |  | <.001 |
| Non | 1,126,564(52.72) | 212,385(68.42) |  | 972,792(50.82) | 366,157(68.68) |  |
| Ex | 412,797(19.32) | 43,591(14.04) |  | 378,607(19.78) | 77,781(14.59) |  |
| Current | 597,595(27.96) | 54,454(17.54) |  | 562,861(29.4) | 89,188(16.73) |  |
| Drinking |  |  | <.001 |  |  | <.001 |
| Non | 1,146,066(53.63) | 225,412(72.61) |  | 990,759(51.76) | 380,719(71.41) |  |
| Mild | 758,298(35.48) | 66,079(21.29) |  | 704,988(36.83) | 119,389(22.39) |  |
| Heavy | 232,592(10.88) | 18,939(6.1) |  | 218,513(11.42) | 33,018(6.19) |  |
| Regular exercise | 446,676(20.9) | 62,904(20.26) | <.001 | 401,641(20.98) | 107,939(20.25) | <.001 |
| Hypertension | 1,152,871(53.95) | 201,324(64.85) | <.001 | 1,016,917(53.12) | 337,278(63.26) | <.001 |
| Dyslipidemia | 854,426(39.98) | 152,464(49.11) | <.001 | 746,892(39.02) | 259,998(48.77) | <.001 |
| CKD | 210,113(9.83) | 49,280(15.87) | <.001 | 181,390(9.48) | 78,003(14.63) | <.001 |
| CVD | 36,591 (1.71) | 10,750 (3.46) | <.001 | 30,340 (1.58) | 17,001 (3.19) | <.001 |
| DM Duration,  ≥5 years | 618,870(28.96) | 121,698(39.2) | <.001 | 544,456(28.44) | 196,112(36.79) | <.001 |
| Insulin | 158,148(7.4) | 41,993(13.53) | <.001 | 136,661(7.14) | 63,480(11.91) | <.001 |
| OHA, ≥3 | 292,639(13.69) | 55,420(17.85) | <.001 | 257,607(13.46) | 90,452(16.97) | <.001 |
| Depression | 184,939(8.65) | 109,611(35.31) | <.001 | 129,878(6.78) | 164,672(30.89) | <.001 |
| Bipolar | 3,737(0.17) | 3,547(1.14) | <.001 | 3,312(0.17) | 3,972(0.75) | <.001 |
| Schizophrenia | 6718(0.31) | 3,594(1.16) | <.001 | 6,328(0.33) | 3,984(0.75) | <.001 |
| Insomnia | - | - | - | 150,697(7.87) | 159,733(29.96) | <.001 |
| Anxiety | 373,393(17.47) | 159,733(51.46) | <.001 | - | - | - |
| BMI | 25.08±3.38 | 24.78±3.4 | <.001 | 25.08±3.39 | 24.92±3.36 | <.001 |
| Waist  Circumference | 85.43±8.62 | 84.82±8.72 | <.001 | 85.48±8.62 | 84.9±8.65 | <.001 |
| SBP | 129.05±15.77 | 128.49±15.86 | <.001 | 129.09±15.77 | 128.59±15.8 | <.001 |
| DBP | 79.27±10.27 | 77.99±10.1 | <.001 | 79.37±10.3 | 78.16±10.05 | <.001 |
| Fasting glucose | 146.42±47.17 | 137.76±45.26 | <.001 | 147.28±47.61 | 138.29±44.14 | <.001 |
| Total cholesterol | 197.45±42.52 | 194.52±43.56 | <.001 | 197.68±42.51 | 194.9±43.13 | <.001 |
| HDL -C | 51.99±23.26 | 52.59±25.32 | <.001 | 51.95±23.13 | 52.48±24.9 | <.001 |
| LDL -C | 111.62±41.11 | 110.45±41.37 | <.001 | 111.61±41.14 | 110.99±41.14 | <.001 |
| eGFR | 86.02±36.3 | 82.18±35.78 | <.001 | 86.32±36.7 | 82.7±34.49 | <.001 |

Units: BMI, kg/m2; waist circumference, cm; SBP, DBP, mmHg; fasting glucose, mg/dL; total cholesterol, HDL-C, and LDL-C, mg/dL; eGFR, mL/min/1.73m².

Abbreviations: Q, quartile; BMI, body mass index; DM, diabetes mellitus; CKD, chronic kidney disease; OHA, oral hypoglycemic agent; SBP, systolic blood pressure; DBP, diastolic blood pressure; HDL-C, high-density lipoprotein cholesterol; LDL-C, low-density lipoprotein cholesterol

**Supplemental Table S3. Discrimination analysis using Harrell’s C model**

|  | Original model | | Mental disorder accumulation model | | | Difference | | | P-value |
| --- | --- | --- | --- | --- | --- | --- | --- | --- | --- |
| Model 1 | - | | 0.5854 (0.5842, 0.5866) | | | - | | | <.0001 |
| Model 2 | 0.7119 (0.7107, 0.7131) | | 0.7192 (0.7180, 0.7204) | | | 0.0073 (0.0061, 0.0085) | | | <.0001 |
| Model 3 | 0.7177 (0.7165, 0.7189) | | 0.7244 (0.7232, 0.7256) | | | 0.0067 (0.0055, 0.0079) | | | <.0001 |
| Model 4 | 0.7385 (0.7373, 0.7397) | | 0.7429 (0.7417, 0.7441) | | | 0.0044 (0.0032, 0.0056) | | | <.0001 |
| Model 5 | 0.7395 (0.7383, 0.7407) | | 0.7438 (0.7426, 0.7450) | | | 0.0043 (0.0031, 0.0055) | | | <.0001 |

Model 1: crude model.

Model 2: age, sex

Model 3: age, sex, BMI, low income, smoking, drinking, and regular exercise.

Model 4: Age, sex, BMI, low income, smoking, drinking, regular exercise, hypertension, dyslipidemia, CKD, fasting glucose, duration of DM, insulin use, duration of DM ≥5 years, and use of more than three types of oral hypoglycemic agents.

Model 5: age, sex, BMI, low income, smoking, drinking, regular exercise, hypertension, dyslipidemia, CKD, fasting glucose, duration of DM, insulin use, duration of DM ≥5 years, use of more than three types of oral hypoglycemic agents, and cardiovascular disease

**Supplemental Table S4. Comparison of goodness of feat using Akaike Information Criterion**

|  | Original Model | Mental disorder  accumulation Model |
| --- | --- | --- |
| Model 1 | 4455259.9 | 4437029.5 |
| Model 2 | 4365859.1 | 4359706.7 |
| Model 3 | 4361177.9 | 4355257 |
| Model 4 | 4341230 | 4336801.7 |
| Model 5 | 4340084.8 | 4335816.8 |

Abbreviation: AIC, Akaike Information Criterion

Model 1: crude model.

Model 2: age, sex

Model 3: age, sex, BMI, low income, smoking, drinking, and regular exercise.

Model 4: Age, sex, BMI, low income, smoking, drinking, regular exercise, hypertension, dyslipidemia, CKD, fasting glucose, duration of DM, insulin use, duration of DM ≥5 years, and use of more than three types of oral hypoglycemic agents.

Model 5: age, sex, BMI, low income, smoking, drinking, regular exercise, hypertension, dyslipidemia, CKD, fasting glucose, duration of DM, insulin use, duration of DM ≥5 years, use of more than three types of oral hypoglycemic agents, and cardiovascular disease

**Supplemental Table S5. Reclassification improvement analysis**

|  | Correctly reclassification, n(%) | | NRI (95% C.I) | P-value | IDI (95% C.I) | P-value |
| --- | --- | --- | --- | --- | --- | --- |
|  | Non-event | Event |  |  |  |  |
| Model 1 | 1577057 (68.8) | 71363 (46.03) | 0.1482 (0.1457, 0.1508) | <.0001 | 0.0074 (0.0072, 0.0075) | <.0001 |
| Model 2 | 1572703 (68.61) | 70832 (45.69) | 0.0252 (0.0232, 0.0271) | <.0001 | 0.0025 (0.0024, 0.0026) | <.0001 |
| Model 3 | 1571154 (68.54) | 71055 (45.83) | 0.0206 (0.0187, 0.0226) | <.0001 | 0.0025 (0.0024, 0.0025) | <.0001 |
| Model 4 | 1565544 (68.29) | 69705 (44.96) | 0.0142 (0.0125, 0.0159) | <.0001 | 0.0018 (0.0017, 0.0019) | <.0001 |
| Model 5 | 1567688 (68.39) | 69576 (44.88) | 0.0133 (0.0116, 0.0150) | <.0001 | 0.0018 (0.0017, 0.0018) | <.0001 |


Abbreviations: IDI, Integrated Discrimination Index; NRI, Net Reclassification Index; CI, confidence interval.

**Supplemental Table S6. Risk of heart failure according to mental disorders**

|  | N | HF | Duration | Rate | Competing risk model 1 | Competing risk model 2 | Competing risk model 3 | Competing risk model 4 | Competing risk model 5 |
| --- | --- | --- | --- | --- | --- | --- | --- | --- | --- |
| **Depression** |  |  |  |  |  |  |  |  |  |
| No | 2,152,836 | 124,708 | 14,675,783.25 | 8.4975 | 1 (Ref.) | 1 (Ref.) | 1 (Ref.) | 1 (Ref.) | 1 (Ref.) |
| Yes | 294,550 | 30,330 | 1,915,358.78 | 15.8352 | 1.835 (1.812, 1.858) | 1.430 (1.412, 1.449) | 1.421 (1.403, 1.440) | 1.328 (1.311, 1.346) | 1.321 (1.304, 1.338) |
| **Bipolar** |  |  |  |  |  |  |  |  |  |
| No | 2,440,102 | 154,382 | 16,544,302.64 | 9.3314 | 1 (Ref.) | 1 (Ref.) | 1 (Ref.) | 1 (Ref.) | 1 (Ref.) |
| Yes | 7,284 | 656 | 46,839.39 | 14.0053 | 1.496 (1.386, 1.614) | 1.515 (1.402, 1.637) | 1.457 (1.349, 1.574) | 1.417 (1.311, 1.532) | 1.406 (1.301, 1.520) |
| **Schizophrenia** |  |  |  |  |  |  |  |  |  |
| No | 2,437,074 | 154,346 | 16,525,979.49 | 9.3396 | 1 (Ref.) | 1 (Ref.) | 1 (Ref.) | 1 (Ref.) | 1 (Ref.) |
| Yes | 10,312 | 692 | 65,162.54 | 10.6196 | 1.149 (1.066, 1.238) | 1.495 (1.386, 1.613) | 1.392 (1.291, 1.502) | 1.409 (1.306, 1.521) | 1.405 (1.302, 1.516) |
| **Insomnia** |  |  |  |  |  |  |  |  |  |
| No | 2,136,956 | 122,917 | 14,580,227.03 | 8.4304 | 1 (Ref.) | 1 (Ref.) | 1 (Ref.) | 1 (Ref.) | 1 (Ref.) |
| Yes | 310,430 | 32,121 | 2,010,915.00 | 15.9733 | 1.860 (1.837, 1.883) | 1.383 (1.366, 1.401) | 1.374 (1.356, 1.391) | 1.311 (1.294, 1.328) | 1.305 (1.289, 1.322) |
| **Anxiety** |  |  |  |  |  |  |  |  |  |
| No | 1,914,260 | 105,420 | 13,062,447.22 | 8.0705 | 1 (Ref.) | 1 (Ref.) | 1 (Ref.) | 1 (Ref.) | 1 (Ref.) |
| Yes | 533,126 | 49,618 | 3,528,694.81 | 14.0613 | 1.726 (1.708, 1.745) | 1.347 (1.332, 1.362) | 1.342 (1.327, 1.357) | 1.293 (1.279, 1.307) | 1.287 (1.273, 1.302) |

The risk of HF development was expressed as HR with 95% confidence interval (CI).

The incidence rate was calculated per 1000 person-years.

Competing risk model 1: crude model.

Competing risk model 2: age, sex

Competing risk model 3: age, sex, BMI, low income, smoking, drinking, and regular exercise.

Competing risk model 4: Age, sex, BMI, low income, smoking, drinking, regular exercise, hypertension, dyslipidemia, CKD, fasting glucose, duration of DM, insulin use, duration of DM ≥5 years, and use of more than three types of oral hypoglycemic agents.

Competing risk model 5: age, sex, BMI, low income, smoking, drinking, regular exercise, hypertension, dyslipidemia, CKD, fasting glucose, duration of DM, insulin use, duration of DM ≥5 years, use of more than three types of oral hypoglycemic agents, and cardiovascular disease

Abbreviations: HF, heart failure; Ref, reference; CKD, chronic kidney disease; DM, diabetes mellitus; BMI, body mass index; HR, hazard ratio

**Supplemental Table S7. Baseline characteristics of subgroups**

|  | Age groups | | | | Sex | | | Income | | |
| --- | --- | --- | --- | --- | --- | --- | --- | --- | --- | --- |
|  | <40 | 40-64 | ≥65 | p-value | Male | Female | p-value | Q2-4 | Q1 | p-value |
| n | 195,384 | 156,0881 | 691,121 |  | 1,496,146 | 951,240 |  | 1,933,929 | 513,457 |  |
| Age, years | 33.71±4.28 | 53.29±6.83 | 71.47±4.95 | <.0001 | 54.77±12.05 | 60.14±11.76 | <.0001 | 56.86±12.37 | 56.88±11.67 | 0.2474 |
| Age group, years |  |  |  |  |  |  | <.0001 |  |  | <.0001 |
| <40 |  |  |  |  | 159,667 (10.67) | 357,17 (3.75) |  | 160253 (8.29) | 35131 (6.84) |  |
| 40-64 |  |  |  |  | 1,004,169 (67.12) | 556,712 (58.52) |  | 1,218,078 (62.98) | 342,803 (66.76) |  |
| ≥65 |  |  |  |  | 332,310 (22.21) | 358,811 (37.72) |  | 555,598 (28.73) | 135,523 (26.39) |  |
| Sex |  |  |  | <.0001 |  |  |  |  |  | <.0001 |
| Men | 159,667 (81.72) | 1,004,169 (64.33) | 332,310 (48.08) |  |  |  |  | 1,207,640 (62.44) | 288,506 (56.19) |  |
| Women | 35,717 (18.28) | 556,712 (35.67) | 358,811 (51.92) |  |  |  |  | 726,289 (37.56) | 224,951 (43.81) |  |
| Income, Q1 (Lowest) | 35,131 (17.98) | 342,803 (21.96) | 135,523 (19.61) | <.0001 | 288,506 (19.28) | 224,951 (23.65) | <.0001 |  |  |  |
| Smoking |  |  |  | <.0001 |  |  | <.0001 |  |  | <.0001 |
| Non | 67,562 (34.58) | 783,867 (50.22) | 487,520 (70.54) |  | 438,658 (29.32) | 900,291 (94.64) |  | 1,046,311 (54.1) | 292,638 (56.99) |  |
| Ex | 25,914 (13.26) | 313,145 (20.06) | 117,329 (16.98) |  | 441,928 (29.54) | 14,460 (1.52) |  | 375,435 (19.41) | 80,953 (15.77) |  |
| Current | 101,908 (52.16) | 463,869 (29.72) | 86,272 (12.48) |  | 615,560 (41.14) | 36,489 (3.84) |  | 512,183 (26.48) | 139,866 (27.24) |  |
| Drinking |  |  |  | <.0001 |  |  | <.0001 |  |  | <.0001 |
| Non | 61,691 (31.57) | 787,439 (50.45) | 522,348 (75.58) |  | 548,850 (36.68) | 822,628 (86.48) |  | 1,067,915 (55.22) | 303,563 (59.12) |  |
| Mild | 105,409 (53.95) | 583,748 (37.4) | 135,220 (19.57) |  | 703,419 (47.02) | 120,958 (12.72) |  | 662,036 (34.23) | 162,341 (31.62) |  |
| Heavy | 28,284 (14.48) | 189,694 (12.15) | 33,553 (4.85) |  | 243,877 (16.3) | 7,654 (0.8) |  | 203,978 (10.55) | 47,553 (9.26) |  |
| Regular exercise | 28,016 (14.34) | 337,617 (21.63) | 143,947 (20.83) | <.0001 | 343,130 (22.93) | 166,450 (17.5) | <.0001 | 409,429 (21.17) | 100,151 (19.51) | <.0001 |
| Hypertension | 47,247 (24.18) | 802,297 (51.4) | 504,651 (73.02) | <.0001 | 794,279 (53.09) | 559,916 (58.86) | <.0001 | 1,065,679 (55.1) | 288,516 (56.19) | <.0001 |
| Dyslipidemia | 47,325 (24.22) | 655,927 (42.02) | 303,638 (43.93) | <.0001 | 532,675 (35.6) | 474,215 (49.85) | <.0001 | 797,486 (41.24) | 209,404 (40.78) | <.0001 |
| CKD | 5,390 (2.76) | 106,230 (6.81) | 147,773 (21.38) | <.0001 | 123,281 (8.24) | 136,112 (14.31) | <.0001 | 206,183 (10.66) | 53,210 (10.36) | <.0001 |
| CVD | 454 (0.23) | 22,109 (1.42) | 24,778 (3.59) | <.0001 | 28,937 (1.93) | 18,404 (1.93) | 0.9719 | 37,340 (1.93) | 10,001 (1.95) | 0.4319 |
| DM Duration, ≥5 years | 10,301 (5.27) | 422,708 (27.08) | 307,559 (44.5) | <.0001 | 407,219 (27.22) | 333,349 (35.04) | <.0001 | 588,373 (30.42) | 152,195 (29.64) | <.0001 |
| Insulin | 7,266 (3.72) | 114,925 (7.36) | 77,950 (11.28) | <.0001 | 108,148 (7.23) | 91,993 (9.67) | <.0001 | 157,467 (8.14) | 42,674 (8.31) | <.0001 |
| OHA, ≥3 types | 8,020 (4.1) | 215,890 (13.83) | 124,149 (17.96) | <.0001 | 193,113 (12.91) | 154,946 (16.29) | <.0001 | 273,388 (14.14) | 74,671 (14.54) | <.0001 |
| Depression | 6,828 (3.49) | 160,737 (10.3) | 126,985 (18.37) | <.0001 | 122,267 (8.17) | 172,283 (18.11) | <.0001 | 230,078 (11.9) | 64,472 (12.56) | <.0001 |
| Bipolar | 369 (0.19) | 4,903 (0.31) | 2,012 (0.29) | <.0001 | 3,532 (0.24) | 3,752 (0.39) | <.0001 | 5,279 (0.27) | 2,005 (0.39) | <.0001 |
| Schizophrenia | 663 (0.34) | 8,016 (0.51) | 1,633 (0.24) | <.0001 | 5,247 (0.35) | 5,065 (0.53) | <.0001 | 6,005 (0.31) | 4,307 (0.84) | <.0001 |
| Insomnia | 6,689 (3.42) | 162,672 (10.42) | 141,069 (20.41) | <.0001 | 129,247 (8.64) | 181,183 (19.05) | <.0001 | 240,499 (12.44) | 69,931 (13.62) | <.0001 |
| Anxiety | 14,403 (7.37) | 297,410 (19.05) | 221,313 (32.02) | <.0001 | 226,756 (15.16) | 306,370 (32.21) | <.0001 | 417,947 (21.61) | 115,179 (22.43) | <.0001 |
| BMI, kg/m2 | 25.8±4.38 | 25.18±3.29 | 24.54±3.2 | <.0001 | 24.97±3.22 | 25.16±3.63 | <.0001 | 25.06±3.36 | 24.98±3.5 | <.0001 |
| Waist Circumference, cm | 85.38±10.73 | 85.27±8.46 | 85.52±8.35 | <.0001 | 86.89±8.03 | 82.93±8.98 | <.0001 | 85.45±8.56 | 85±8.89 | <.0001 |
| SBP, mmHg | 126.28±14.6 | 128.1±15.53 | 131.73±16.28 | <.0001 | 129.3±15.39 | 128,.47±16.36 | <.0001 | 128.94±15.66 | 129.12±16.2 | <.0001 |
| DBP, mmHg | 79.43±10.44 | 79.56±10.24 | 78±10.16 | <.0001 | 79.92±10.24 | 77.84±10.17 | <.0001 | 79.09±10.22 | 79.17±10.41 | <.0001 |
| Fasting glucose, mg/dL | 156.11±52.02 | 148.43±47.64 | 135.26±42.21 | <.0001 | 148.13±48.03 | 140.9±45.04 | <.0001 | 144.84±46.14 | 147.13±50.15 | <.0001 |
| Total cholesterol, mg/dL | 202.38±42.44 | 199.03±42.89 | 191.16±41.58 | <.0001 | 194.44±41.94 | 201.22±43.45 | <.0001 | 196.94±42.57 | 197.61±43.01 | <.0001 |
| HDL-C, mg/dL | 51.86±21.57 | 52.24±23.42 | 51.72±24.3 | <.0001 | 50.71±22.99 | 54.19±24.21 | <.0001 | 51.96±23.91 | 52.45±22.03 | <.0001 |
| LDL-C, mg/dL | 111.08±41.48 | 112.3±41.75 | 109.73±39.58 | <.0001 | 107.91±40.92 | 117.08±40.86 | <.0001 | 111.45±41.03 | 111.57±41.56 | 0.0771 |
| eGFR | 95.45±43.64 | 87.8±36.07 | 77.6±32.82 | <.0001 | 86.9±39.3 | 83.38±30.76 | <.0001 | 85.4±36.72 | 86.01±34.48 | <.0001 |

**Supplemental Table S7. Baseline characteristics of subgroups- continued**

|  | BMI | | | Smoking | | | Drinking | | |
| --- | --- | --- | --- | --- | --- | --- | --- | --- | --- |
|  | <25 | ≥25 | p-value | Non, Ex | Current | p-value | Non, Mild | Heavy | p-value |
| n | 1,261,492 | 1,185,894 |  | 1,795,337 | 652,049 |  | 2,195,855 | 251,531 |  |
| Age, years | 57.91±12.34 | 55.74±11.99 | <.0001 | 58.9±11.83 | 51.26±11.52 | <.0001 | 57.39±12.26 | 52.28±10.84 | <.0001 |
| Age group, years |  |  | <.0001 |  |  | <.0001 |  |  | <.0001 |
| <40 | 88,068 (6.98) | 107,316 (9.05) |  | 93,476 (5.21) | 101,908 (15.63) |  | 167,100 (7.61) | 28,284 (11.24) |  |
| 40-64 | 778,034 (61.68) | 782,847 (66.01) |  | 1,097,012 (61.1) | 463,869 (71.14) |  | 1,371,187 (62.44) | 189,694 (75.42) |  |
| ≥65 | 395390 (31.34) | 295,731 (24.94) |  | 604,849 (33.69) | 86,272 (13.23) |  | 657,568 (29.95) | 33,553 (13.34) |  |
| Sex |  |  | <.0001 |  |  | <.0001 |  |  | <.0001 |
| Men | 775,207 (61.45) | 720,939 (60.79) |  | 880,586 (49.05) | 615,560 (94.4) |  | 1,252,269 (57.03) | 243,877 (96.96) |  |
| Women | 486,285 (38.55) | 464,955 (39.21) |  | 914,751 (50.95) | 36,489 (5.6) |  | 943,586 (42.97) | 7,654 (3.04) |  |
| Income, Q1 (Lowest) | 269,702 (21.38) | 243,755 (20.55) | <.0001 | 373,591 (20.81) | 139,866 (21.45) | <.0001 | 465,904 (21.22) | 47,553 (18.91) | <.0001 |
| Smoking |  |  | <.0001 |  |  | <.0001 |  |  | <.0001 |
| Non | 685,875 (54.37) | 653,074 (55.07) |  | 1,338,949 (74.58) | 0 (0) |  | 1,293,695 (58.92) | 45,254 (17.99) |  |
| Ex | 222,806 (17.66) | 233,582 (19.7) |  | 456,388 (25.42) | 0 (0) |  | 386,231 (17.59) | 70,157 (27.89) |  |
| Current | 352,811 (27.97) | 299,238 (25.23) |  | 0 (0) | 652,049 (100) |  | 515,929 (23.5) | 136,120 (54.12) |  |
| Drinking |  |  | <.0001 |  |  | <.0001 |  |  | <.0001 |
| Non | 718,031 (56.92) | 653,447 (55.1) |  | 1,193,547 (66.48) | 177,931 (27.29) |  | 1,371,478 (62.46) | 0 (0) |  |
| Mild | 421,835 (33.44) | 402,542 (33.94) |  | 486,379 (27.09) | 337,998 (51.84) |  | 824,377 (37.54) | 0 (0) |  |
| Heavy | 121,626 (9.64) | 129,905 (10.95) |  | 115,411 (6.43) | 136,120 (20.88) |  | 0 (0) | 251,531 (100) |  |
| Regular exercise | 270,605 (21.45) | 238,975 (20.15) | <.0001 | 390,227 (21.74) | 119,353 (18.3) | <.0001 | 455,117 (20.73) | 54,463 (21.65) | <.0001 |
| Hypertension | 615,129 (48.76) | 739,066 (62.32) | <.0001 | 1,049,787 (58.47) | 304,408 (46.68) | <.0001 | 1,213,084 (55.24) | 141,111 (56.1) | <.0001 |
| Dyslipidemia | 464,158 (36.79) | 542,732 (45.77) | <.0001 | 778,298 (43.35) | 228,592 (35.06) | <.0001 | 917,976 (41.8) | 88,914 (35.35) | <.0001 |
| CKD | 133,117 (10.55) | 126,276 (10.65) | 0.0149 | 219,542 (12.23) | 39,851 (6.11) | <.0001 | 246,557 (11.23) | 12,836 (5.1) | <.0001 |
| CVD | 25,,464 (2.02) | 21877 (1.84) | <.0001 | 37,685 (2.1) | 9,656 (1.48) | <.0001 | 4,443 (2.02) | 2,902 (1.16) | <.0001 |
| DM Duration, ≥5 years | 420,052 (33.3) | 320,516 (27.03) | <.0001 | 592,112 (32.98) | 148,456 (22.77) | <.0001 | 686,233 (31.25) | 54,335 (21.6) | <.0001 |
| Insulin | 115,696 (9.17) | 84,445 (7.12) | <.0001 | 158,200 (8.81) | 41,941 (6.43) | <.0001 | 187,340 (8.53) | 12,801 (5.09) | <.0001 |
| OHA, ≥3 types | 189,359 (15.01) | 158,700 (13.38) | <.0001 | 268,718 (14.97) | 79,341 (12.17) | <.0001 | 321,028 (14.62) | 27,031 (10.75) | <.0001 |
| Depression | 158,417 (12.56) | 136,133 (11.48) | <.0001 | 245,016 (13.65) | 49,534 (7.6) | <.0001 | 277,906 (12.66) | 16,644 (6.62) | <.0001 |
| Bipolar | 3,251 (0.26) | 4,033 (0.34) | <.0001 | 5,335 (0.3) | 1,949 (0.3) | 0.8246 | 6,940 (0.32) | 344 (0.14) | <.0001 |
| Schizophrenia | 4,502 (0.36) | 5,810 (0.49) | <.0001 | 7,197 (0.4) | 3,115 (0.48) | <.0001 | 9,957 (0.45) | 355 (0.14) | <.0001 |
| Insomnia | 169,921 (13.47) | 140,509 (11.85) | <.0001 | 255,976 (14.26) | 54,454 (8.35) | <.0001 | 291,491 (13.27) | 18,939 (7.53) | <.0001 |
| Anxiety | 282,280 (22.38) | 250,846 (21.15) | <.0001 | 443,938 (24.73) | 89,188 (13.68) | <.0001 | 500,108 (22.78) | 33,018 (13.13) | <.0001 |
| BMI, kg/m2 | 22.52±1.8 | 27.74±2.49 | <.0001 | 25.12±3.37 | 24.83±3.43 | <.0001 | 25.03±3.39 | 25.22±3.33 | <.0001 |
| Waist Circumference, cm | 80.37±6.61 | 90.66±7.26 | <.0001 | 85.04±8.7 | 86.2±8.39 | <.0001 | 85.11±8.65 | 87.48±8.18 | <.0001 |
| SBP, mmHg | 127.04±15.88 | 131.04±15.41 | <.0001 | 129.27±15.91 | 128.19±15.4 | <.0001 | 128.66±15.75 | 131.74±15.73 | <.0001 |
| DBP, mmHg | 77.67±10.1 | 80.64±10.21 | <.0001 | 78.88±10.22 | 79.73±10.33 | <.0001 | 78.78±10.19 | 81.99±10.44 | <.0001 |
| Fasting glucose, mg/dL | 146.09±49.72 | 144.51±43.95 | <.0001 | 142.78±45.25 | 152.33±50.92 | <.0001 | 144.35±46.76 | 153.79±48.39 | <.0001 |
| Total cholesterol, mg/dL | 194±42.18 | 200.35±42.93 | <.0001 | 196.67±42.6 | 198.21±42.81 | <.0001 | 196.75±42.51 | 199.91±43.9 | <.0001 |
| HDL-C, mg/dL | 53.53±24.55 | 50.51±22.29 | <.0001 | 52.59±23.83 | 50.61±22.61 | <.0001 | 51.77±23.32 | 54.61±25.17 | <.0001 |
| LDL-C, mg/dL | 110.14±40.59 | 112.9±41.68 | <.0001 | 112.63±40.68 | 108.28±42.21 | <.0001 | 112.44±40.77 | 103.07±43.35 | <.0001 |
| eGFR | 85.99±36.24 | 85.04±36.28 | <.0001 | 84.01±35.09 | 89.71±39.01 | <.0001 | 84.9±35.9 | 90.99±38.86 | <.0001 |

**Supplemental Table S7. Baseline characteristics of subgroups- continued**

|  | Regular exercise | | | Hypertension | | | Dyslipidemia | | |
| --- | --- | --- | --- | --- | --- | --- | --- | --- | --- |
|  | No | Yes | p-value | No | Yes | p-value | No | Yes | p-value |
| n | 1,937,806 | 509,580 |  | 1,093,191 | 1,354,195 |  | 1,440,496 | 1,006,890 |  |
| Age, years | 56.66±12.51 | 57.62±11.02 | <.0001 | 52.62±12.12 | 60.28±11.19 | <.0001 | 55.87±12.82 | 58.28±11.15 | <.0001 |
| Age group, years |  |  | <.0001 |  |  | <.0001 |  |  | <.0001 |
| <40 | 167,368 (8.64) | 28,016 (5.5) |  | 148,137 (13.55) | 47,247 (3.49) |  | 148,059 (10.28) | 47,325 (4.7) |  |
| 40-64 | 1,223,264 (63.13) | 337,617 (66.25) |  | 758,584 (69.39) | 802,297 (59.25) |  | 904,954 (62.82) | 655,927 (65.14) |  |
| ≥65 | 547,174 (28.24) | 143,947 (28.25) |  | 186,470 (17.06) | 504,651 (37.27) |  | 387,483 (26.9) | 303,638 (30.16) |  |
| Sex |  |  | <.0001 |  |  | <.0001 |  |  | <.0001 |
| Men | 1,153,016 (59.5) | 343,130 (67.34) |  | 701,867 (64.2) | 794,279 (58.65) |  | 96,3471 (66.88) | 532,675 (52.9) |  |
| Women | 784,790 (40.5) | 166,450 (32.66) |  | 391,324 (35.8) | 559,916 (41.35) |  | 477,025 (33.12) | 474,215 (47.1) |  |
| Income, Q1 (Lowest) | 413,306 (21.33) | 100,151 (19.65) | <.0001 | 224,941 (20.58) | 288,516 (21.31) | <.0001 | 304,053 (21.11) | 209,404 (20.8) | <.0001 |
| Smoking |  |  | <.0001 |  |  | <.0001 |  |  | <.0001 |
| Non | 1,076,756 (55.57) | 262,193 (51.45) |  | 553,481 (50.63) | 785,468 (58) |  | 742,116 (51.52) | 596,833 (59.27) |  |
| Ex | 328,354 (16.94) | 128,034 (25.13) |  | 192,069 (17.57) | 264,319 (19.52) |  | 274,923 (19.09) | 181,465 (18.02) |  |
| Current | 532,696 (27.49) | 119,353 (23.42) |  | 347,641 (31.8) | 304,408 (22.48) |  | 423,457 (29.4) | 228,592 (22.7) |  |
| Drinking |  |  | <.0001 |  |  | <.0001 |  |  | <.0001 |
| Non | 1,105,366 (57.04) | 266,112 (52.22) |  | 578,506 (52.92) | 792,972 (58.56) |  | 750,767 (52.12) | 620,711 (61.65) |  |
| Mild | 635,372 (32.79) | 189,005 (37.09) |  | 404,265 (36.98) | 420,112 (31.02) |  | 527,112 (36.59) | 297,265 (29.52) |  |
| Heavy | 197,068 (10.17) | 54,463 (10.69) |  | 110,420 (10.1) | 141,111 (10.42) |  | 162,617 (11.29) | 88,914 (8.83) |  |
| Regular exercise |  | | | 221,199 (20.23) | 288,381 (21.3) | <.0001 | 299,463 (20.79) | 210,117 (20.87) | 0.134 |
| Hypertension | 1,065,814 (55) | 288,381 (56.59) | <.0001 |  | | | 708,050 (49.15) | 646,145 (64.17) | <.0001 |
| Dyslipidemia | 796,773 (41.12) | 210,117 (41.23) | 0.134 | 360,475 (33) | 646,145 (47.71) | <.0001 |  | | |
| CKD | 206,992 (10.68) | 52,401 (10.28) | <.0001 | 66,289 (6.06) | 193,104 (14.26) | <.0001 | 127,501 (8.85) | 131,892 (13.1) | <.0001 |
| CVD | 38,030 (1.96) | 9,311 (1.83) | <.0001 | 9,710 (0.89) | 37,631 (2.78) | <.0001 | 16,044 (1.11) | 31,297 (3.11) | <.0001 |
| DM Duration, ≥5 years | 566,760 (29.25) | 173,808 (34.11) | <.0001 | 254,747 (23.3) | 485,821 (35.88) | <.0001 | 388,143 (26.95) | 352,425 (35) | <.0001 |
| Insulin | 158,260 (8.17) | 41,881 (8.22) | 0.2302 | 66,500 (6.08) | 133,641 (9.87) | <.0001 | 95,374 (6.62) | 104,767 (10.41) | <.0001 |
| OHA, ≥3 types | 273,479 (14.11) | 74,580 (14.64) | <.0001 | 125,449 (11.48) | 222,610 (16.44) | <.0001 | 165,389 (11.48) | 182,670 (18.14) | <.0001 |
| Depression | 236,098 (12.18) | 58,452 (11.47) | <.0001 | 106,080 (9.7) | 188,470 (13.92) | <.0001 | 144,799 (10.05) | 149,751 (14.87) | <.0001 |
| Bipolar | 5,910 (0.3) | 1,374 (0.27) | <.0001 | 3,600 (0.33) | 3,684 (0.27) | <.0001 | 3,881 (0.27) | 3,403 (0.34) | <.0001 |
| Schizophrenia | 8,776 (0.45) | 1,536 (0.3) | <.0001 | 5,938 (0.54) | 4,374 (0.32) | <.0001 | 5,974 (0.41) | 4,338 (0.43) | 0.0555 |
| Insomnia | 247,526 (12.77) | 62,904 (12.34) | <.0001 | 109,106 (9.98) | 201,324 (14.87) | <.0001 | 157,966 (10.97) | 152,464 (15.14) | <.0001 |
| Anxiety | 425,187 (21.94) | 107,939 (21.18) | <.0001 | 195,848 (17.92) | 337,278 (24.91) | <.0001 | 273,128 (18.96) | 259,998 (25.82) | <.0001 |
| BMI, kg/m2 | 25.08±3.45 | 24.92±3.15 | <.0001 | 24.43±3.25 | 25.54±3.41 | <.0001 | 24.73±3.4 | 25.5±3.32 | <.0001 |
| Waist Circumference, cm | 85.43±8.74 | 85.07±8.22 | <.0001 | 83.5±8.42 | 86.85±8.51 | <.0001 | 84.73±8.68 | 86.25±8.48 | <.0001 |
| SBP, mmHg | 128.99±15.86 | 128.94±15.47 | 0.068 | 121.22±10.68 | 135.24±16.43 | <.0001 | 128.63±15.75 | 129.48±15.81 | <.0001 |
| DBP, mmHg | 79.18±10.31 | 78.83±10.07 | <.0001 | 75.22±7.56 | 82.25±11.04 | <.0001 | 79.09±10.24 | 79.14±10.28 | <.0001 |
| Fasting glucose, mg/dL | 146.22±47.84 | 141.91±43.58 | <.0001 | 149.35±48.27 | 142.07±45.73 | <.0001 | 145.76±45.42 | 144.69±49.21 | <.0001 |
| Total cholesterol, mg/dL | 198.01±42.93 | 193.52±41.43 | <.0001 | 199.86±41.97 | 194.83±43.08 | <.0001 | 189.3±28.41 | 208.21±55.31 | <.0001 |
| HDL-C, mg/dL | 51.99±23.89 | 52.35±22.09 | <.0001 | 52.49±23.17 | 51.72±23.81 | <.0001 | 51.22±22.34 | 53.28±25.09 | <.0001 |
| LDL-C, mg/dL | 111.97±41.39 | 109.61±40.13 | <.0001 | 114.51±40.52 | 109.02±41.47 | <.0001 | 107.01±32.35 | 117.86±50.48 | <.0001 |
| eGFR | 85.71±36.38 | 84.85±35.8 | <.0001 | 88.82±36.03 | 82.87±36.23 | <.0001 | 86.93±36.42 | 83.53±35.94 | <.0001 |

**Supplemental Table S7. Baseline characteristics of subgroups- continued**

|  | CKD | | | DM Duration | | | CVD | | |
| --- | --- | --- | --- | --- | --- | --- | --- | --- | --- |
|  | No | Yes | p-value | <5 years | ≥5 years | p-value | No | Yes | p-value |
| n | 2,187,993 | 259,393 |  | 1,706,818 | 740,568 |  | 2,400,045 | 47,341 |  |
| Age, years | 55.89±11.99 | 65.06±11 | <.0001 | 54.7±12.38 | 61.84±10.25 | <.0001 | 56.71±12.21 | 64.39±10.23 | <.0001 |
| Age group, years |  |  | <.0001 |  |  | <.0001 |  |  | <.0001 |
| <40 | 189,994 (8.68) | 5,390 (2.08) |  | 185,083 (10.84) | 10,301 (1.39) |  | 194,930  (8.12) | 454  (0.96) |  |
| 40-64 | 1,454,651 (66.48) | 106,230 (40.95) |  | 1,138,173 (66.68) | 422,708 (57.08) |  | 1,538,772  (64.11) | 22,109  (46.7) |  |
| ≥65 | 543,348 (24.83) | 147,773 (56.97) |  | 383,562 (22.47) | 307,559 (41.53) |  | 666,343  (27.76) | 24,778  (52.34) |  |
| Sex |  |  | <.0001 |  |  | <.0001 |  |  | 0.9719 |
| Men | 1,372,865 (62.75) | 123,281 (47.53) |  | 1,088,927 (63.8) | 407,219 (54.99) |  | 1,467,209  (61.13) | 28,937  (61.12) |  |
| Women | 815,128 (37.25) | 136,112 (52.47) |  | 617891 (36.2) | 333,349 (45.01) |  | 932,836  (38.87) | 18,404  (38.88) |  |
| Income, Q1 (Lowest) | 460,247 (21.04) | 53,210 (20.51) | <.0001 | 361,262 (21.17) | 152,195 (20.55) | <.0001 | 503,456  (20.98) | 10,001  (21.13) | 0.4319 |
| Smoking |  |  | <.0001 |  |  | <.0001 |  |  | <.0001 |
| Non | 1,163,873 (53.19) | 175,076 (67.49) |  | 882,914 (51.73) | 456,035 (61.58) |  | 1,311,785  (54.66) | 27,164  (57.38) |  |
| Ex | 411,922 (18.83) | 44,466 (17.14) |  | 320,311 (18.77) | 136,077 (18.37) |  | 445,867  (18.58) | 10,521  (22.22) |  |
| Current | 612,198 (27.98) | 39,851 (15.36) |  | 503593 (29.5) | 148,456 (20.05) |  | 642,393  (26.77) | 9,656  (20.4) |  |
| Drinking |  |  | <.0001 |  |  | <.0001 |  |  | <.0001 |
| Non | 1,181,855 (54.02) | 189,623 (73.1) |  | 877,560 (51.41) | 493,918 (66.69) |  | 1,337,743  (55.74) | 33,735  (71.26) |  |
| Mild | 767,443 (35.08) | 56,934 (21.95) |  | 632,062 (37.03) | 192,315 (25.97) |  | 813,680  (33.9) | 10,697  (22.6) |  |
| Heavy | 238,695 (10.91) | 12,836 (4.95) |  | 197,196 (11.55) | 54,335 (7.34) |  | 248,622  (10.36) | 2,909  (6.14) |  |
| Regular exercise | 457,179 (20.89) | 52,401 (20.2) | <.0001 | 335,772 (19.67) | 173,808 (23.47) | <.0001 | 500,269  (20.84) | 9,311  (19.67) | <.0001 |
| Hypertension | 1,161,091 (53.07) | 193,104 (74.44) | <.0001 | 868,374 (50.88) | 485,821 (65.6) | <.0001 | 1,316,564  (54.86) | 37,631  (79.49) | <.0001 |
| Dyslipidemia | 874,998 (39.99) | 131,892 (50.85) | <.0001 | 654,465 (38.34) | 352,425 (47.59) | <.0001 | 975,593  (40.65) | 31,297  (66.11) | <.0001 |
| CKD |  | | | 138,589 (8.12) | 120,804 (16.31) | <.0001 | 249,916  (10.41) | 9,477  (20.02) | <.0001 |
| CVD | 37,864 (1.73) | 9,477 (3.65) | <.0001 | 26,694 (1.56) | 20,647 (2.79) | <.0001 |  | | |
| DM Duration, ≥5 years | 619,764 (28.33) | 120,804 (46.57) | <.0001 |  | | | 719,921 (30) | 20,647 (43.61) | <.0001 |
| Insulin | 159,751 (7.3) | 40,390 (15.57) | <.0001 | 69,066 (4.05) | 131,075 (17.7) | <.0001 | 187,010 (7.79) | 13,131 (27.74) | <.0001 |
| OHA, ≥3 types | 298,539 (13.64) | 49,520 (19.09) | <.0001 | 111,189 (6.51) | 236,870 (31.98) | <.0001 | 336,649 (14.03) | 11,410 (24.1) | <.0001 |
| Depression | 246,929 (11.29) | 47,621 (18.36) | <.0001 | 175,465 (10.28) | 119,085 (16.08) | <.0001 | 283,469 (11.81) | 11,081 (23.41) | <.0001 |
| Bipolar | 6,388 (0.29) | 896 (0.35) | <.0001 | 4,850 (0.28) | 2,434 (0.33) | <.0001 | 7,060 (0.29) | 224 (0.47) | <.0001 |
| Schizophrenia | 9,231 (0.42) | 1,081 (0.42) | 0.7017 | 7,407 (0.43) | 2,905 (0.39) | <.0001 | 10,096 (0.42) | 216 (0.46) | 0.2363 |
| Insomnia | 261,150 (11.94) | 49,280 (19) | <.0001 | 188,732 (11.06) | 121,698 (16.43) | <.0001 | 299,680 (12.49) | 10,750 (22.71) | <.0001 |
| Anxiety | 455,123 (20.8) | 78,003 (30.07) | <.0001 | 337,014 (19.75) | 196,112 (26.48) | <.0001 | 516,125 (21.5) | 17,001 (35.91) | <.0001 |
| BMI, kg/m2 | 25.05±3.39 | 25.03±3.33 | 0.0622 | 25.21±3.45 | 24.66±3.19 | <.0001 | 25.05±3.39 | 24.81±3.26 | <.0001 |
| Waist Circumference, cm | 85.29±8.63 | 85.88±8.62 | <.0001 | 85.43±8.75 | 85.18±8.36 | <.0001 | 85.34±8.64 | 86.12±8.39 | <.0001 |
| SBP, mmHg | 128.77±15.65 | 130.7±16.76 | <.0001 | 128.98±15.71 | 128.99±15.94 | 0.6165 | 128.96±15.76 | 130.04±16.87 | <.0001 |
| DBP, mmHg | 79.2±10.23 | 78.3±10.49 | <.0001 | 79.77±10.31 | 77.59±9.97 | <.0001 | 79.12±10.25 | 78.41±10.61 | <.0001 |
| Fasting glucose, mg/dL | 145.92±46.58 | 140.28±50.3 | <.0001 | 144.03±43.47 | 148.3±54.2 | <.0001 | 145.46±46.96 | 138.28±49.51 | <.0001 |
| Total cholesterol, mg/dL | 197.35±42.37 | 194.78±44.99 | <.0001 | 201.55±42.65 | 186.76±40.86 | <.0001 | 197.34±42.57 | 183.9±45.21 | <.0001 |
| HDL-C, mg/dL | 51.95±20.57 | 53.01±40.67 | <.0001 | 52.49±23.01 | 51.08±24.65 | <.0001 | 52.11±23.49 | 49.87±25.54 | <.0001 |
| LDL-C, mg/dL | 111.57±41.11 | 110.67±41.37 | <.0001 | 114.2±41.58 | 105.2±39.4 | <.0001 | 111.64±41.08 | 103.09±43.58 | <.0001 |
| eGFR | 90.19±35.13 | 46.25±16.45 | <.0001 | 87.05±36.53 | 82.04±35.39 | <.0001 | 85.66±36.21 | 79.15±38.41 | <.0001 |

**Supplemental Table S7. Baseline characteristics of subgroups- continued**

|  | Insulin | | | OHA | | |
| --- | --- | --- | --- | --- | --- | --- |
|  | No | Yes | p-value | <3 | ≥3 | p-value |
| N | 2,247,245 | 200,141 |  | 2,099,327 | 348,059 |  |
| Age, years | 56.53±12.25 | 60.52±11.22 | <.0001 | 56.33±12.4 | 60.06±10.57 | <.0001 |
| Age group, years |  |  | <.0001 |  |  | <.0001 |
| <40 | 188,118 (8.37) | 7,266 (3.63) |  | 187,364 (8.92) | 8,020 (2.3) |  |
| 40-64 | 1,445,956 (64.34) | 114,925 (57.42) |  | 1,344,991 (64.07) | 215,890 (62.03) |  |
| ≥65 | 613,171 (27.29) | 77,950 (38.95) |  | 566,972 (27.01) | 124,149 (35.67) |  |
| Sex |  |  | <.0001 |  |  | <.0001 |
| Men | 1,387,998 (61.76) | 108,148 (54.04) |  | 1,303,033 (62.07) | 193,113 (55.48) |  |
| Women | 859,247 (38.24) | 91,993 (45.96) |  | 796,294 (37.93) | 154,946 (44.52) |  |
| Income, Q1 (Lowest) | 470,783 (20.95) | 42,674 (21.32) | <.0001 | 438,786 (20.9) | 74,671 (21.45) | <.0001 |
| Smoking |  |  | <.0001 |  |  | <.0001 |
| Non | 1,217,194 (54.16) | 121,755 (60.83) |  | 1,132,052 (53.92) | 206,897 (59.44) |  |
| Ex | 419,943 (18.69) | 36,445 (18.21) |  | 394,567 (18.79) | 61,821 (17.76) |  |
| Current | 610,108 (27.15) | 41,941 (20.96) |  | 572,708 (27.28) | 79,341 (22.8) |  |
| Drinking |  |  | <.0001 |  |  | <.0001 |
| Non | 1,228,407 (54.66) | 143,071 (71.49) |  | 1,140,824 (54.34) | 230,654 (66.27) |  |
| Mild | 780,108 (34.71) | 44,269 (22.12) |  | 734,003 (34.96) | 90,374 (25.97) |  |
| Heavy | 238,730 (10.62) | 12,801 (6.4) |  | 224,500 (10.69) | 27,031 (7.77) |  |
| Regular exercise | 467,699 (20.81) | 41,881 (20.93) | 0.2302 | 435,000 (20.72) | 74,580 (21.43) | <.0001 |
| Hypertension | 1,220,554 (54.31) | 133,641 (66.77) | <.0001 | 1,131,585 (53.9) | 222,610 (63.96) | <.0001 |
| Dyslipidemia | 902,123 (40.14) | 104,767 (52.35) | <.0001 | 824,220 (39.26) | 182,670 (52.48) | <.0001 |
| CKD | 219,003 (9.75) | 40,390 (20.18) | <.0001 | 209,873 (10) | 49,520 (14.23) | <.0001 |
| CVD | 34,210 (1.52) | 13,131 (6.56) | <.0001 | 35,931 (1.71) | 11,410 (3.28) | <.0001 |
| DM Duration, ≥5 years | 609,493 (27.12) | 131,075 (65.49) | <.0001 | 503,698 (23.99) | 236,870 (68.05) | <.0001 |
| Insulin |  | | | 143,630 (6.84) | 56,511 (16.24) | <.0001 |
| OHA, ≥3 types | 291,548 (12.97) | 56,511 (28.24) | <.0001 |  | | |
| Depression | 251,087 (11.17) | 43,463 (21.72) | <.0001 | 240,880 (11.47) | 53,670 (15.42) | <.0001 |
| Bipolar | 6,378 (0.28) | 906 (0.45) | <.0001 | 6,034 (0.29) | 1,250 (0.36) | <.0001 |
| Schizophrenia | 9,309 (0.41) | 1,003 (0.5) | <.0001 | 8,652 (0.41) | 1,660 (0.48) | <.0001 |
| Insomnia | 268,437 (11.95) | 41,993 (20.98) | <.0001 | 255,010 (12.15) | 55,420 (15.92) | <.0001 |
| Anxiety | 469,646 (20.9) | 63,480 (31.72) | <.0001 | 442,674 (21.09) | 90,452 (25.99) | <.0001 |
| BMI, kg/m2 | 25.09±3.38 | 24.52±3.43 | <.0001 | 25.08±3.39 | 24.86±3.37 | <.0001 |
| Waist Circumference, cm | 85.39±8.6 | 84.99±8.94 | <.0001 | 85.31±8.64 | 85.62±8.58 | <.0001 |
| SBP, mmHg | 129.07±15.71 | 127.94±16.54 | <.0001 | 129.13±15.79 | 128.08±15.71 | <.0001 |
| DBP, mmHg | 79.29±10.24 | 77.06±10.26 | <.0001 | 79.34±10.3 | 77.72±9.9 | <.0001 |
| Fasting glucose, mg/dL | 144.8±44.52 | 151.24±68.87 | <.0001 | 143.91±44.64 | 153.86±58.68 | <.0001 |
| Total cholesterol, mg/dL | 198.1±42.38 | 185.59±44.07 | <.0001 | 198.62±42.52 | 187.79±42.34 | <.0001 |
| HDL-C, mg/dL | 52.13±23.27 | 51.29±26.28 | <.0001 | 52.31±23.28 | 50.58±24.95 | <.0001 |
| LDL-C, mg/dL | 112.1±41.07 | 104.42±41.25 | <.0001 | 112.5±41.15 | 105.27±40.57 | <.0001 |
| eGFR | 85.95±35.98 | 80.75±39.01 | <.0001 | 85.8±36.28 | 83.88±36.12 | <.0001 |

Continuous variables are expressed as mean ± standard deviation, and categorical variables are expressed as frequency (percent).

Abbreviations: Q, quartile; CKD, chronic kidney disease; CVD, cardiovascular disease; DM, diabetes mellitus; OHA, oral hypoglycemic agent; BMI, body mass index; SBP, systolic blood pressure; DBP, diastolic blood pressure; HDL-C, high-density lipoprotein cholesterol; LDL-C, low-density lipoprotein cholesterol; eGFR, estimated glomerular filtration rate; TG, triglyceride.

**Supplemental Table S8. Follow-up duration and incidence of heart failure in subgroups.**

|  |  | N | Event | Duration | IR |
| --- | --- | --- | --- | --- | --- |
| Age groups | <40 | 195,384 | 2,304 | 1,359,063.80 | 1.69528 |
|  | 40-64 | 1,560,881 | 65,766 | 10,793,544.18 | 6.09309 |
|  | ≥65 | 691,121 | 86,968 | 4,438,534.05 | 19.59386 |
| Sex | Men | 1,496,146 | 88,288 | 10,084,720.62 | 8.75463 |
|  | Women | 951,240 | 66,750 | 6,506,421.40 | 10.2591 |
| Income | Q2-4 | 1,933,929 | 120,680 | 13,149,446.96 | 9.17757 |
|  | Q1 | 513,457 | 34,358 | 3,441,695.07 | 9.98287 |
| BMI | <25 | 1,261,492 | 83,162 | 8,471,619.31 | 9.81654 |
|  | 25+ | 1,185,894 | 71,876 | 8,119,522.72 | 8.85224 |
| Smoking | Non, ex | 1,795,337 | 119,600 | 12,215,804.19 | 9.7906 |
|  | Current | 652,049 | 35,438 | 4,375,337.83 | 8.09949 |
| Drinking | Non, Mild | 2,195,855 | 142,505 | 14,888,248.75 | 9.57164 |
|  | Heavy | 251,531 | 12,533 | 1,702,893.28 | 7.35983 |
| Regular exercise | No | 1,937,806 | 125,432 | 13,076,981.44 | 9.59182 |
|  | Yes | 509,580 | 29,606 | 3,514,160.58 | 8.42477 |
| Hypertension | No | 1,093,191 | 44,600 | 7,507,980.39 | 5.94035 |
|  | Yes | 1,354,195 | 110,438 | 9,083,161.64 | 12.15854 |
| Dyslipidemia | No | 1,440,496 | 83,017 | 9,755,839.20 | 8.50947 |
|  | Yes | 1,006,890 | 72,021 | 6,835,302.83 | 10.53662 |
| CKD | No | 2,187,993 | 121,431 | 14,907,997.89 | 8.14536 |
|  | Yes | 259,393 | 33,607 | 1,683,144.14 | 19.9668 |
| CVD History | No | 2,400,045 | 147,731 | 16,297,679.40 | 9.06454 |
|  | Yes | 47,341 | 7,307 | 293,462.63 | 24.89925 |
| Insulin use | No | 1,706,818 | 128,372 | 15,321,312.06 | 8.37866 |
|  | Yes | 740,568 | 26,666 | 1,269,829.97 | 20.99966 |
| OHA Number | <3 | 2,247,245 | 122,841 | 14,222,685.49 | 8.63698 |
|  | 3+ | 200,141 | 32,197 | 2,368,456.54 | 13.59409 |
| DM Duration | <5 | 2,099,327 | 83,177 | 11,609,864.64 | 7.16434 |
|  | 5+ | 348,059 | 71,861 | 4,981,277.39 | 14.42622 |

Abbreviations: IR, incidence rate; BMI, body mass index; CKD, chronic kidney disease; CVD, cardiovascular disease; OHA, oral hypoglycemic agent; DM, diabetes mellitus.

The incidence rate was calculated per 1000 person-years.

**Supplemental Table S9. Subgroup analysis–Depression**

|  |  | Depression | N | HF | Duration | Rate | Competing risk  Model 5 | * p for interaction |
| --- | --- | --- | --- | --- | --- | --- | --- | --- |
| Age groups | <40 | No | 188556 | 2143 | 1312844.3 | 1.6323 | 1 (Ref.) | <.0001 |
|  |  | Yes | 6828 | 161 | 46219.5 | 3.4834 | 2.085 (1.777, 2.446) |  |
|  | 40-64 | No | 1400144 | 54853 | 9710194.25 | 5.649 | 1 (Ref.) |  |
|  |  | Yes | 160737 | 10913 | 1083349.93 | 10.0734 | 1.526 (1.495, 1.559) |  |
|  | ≥65 | No | 564136 | 67712 | 3652744.7 | 18.5373 | 1 (Ref.) |  |
|  |  | Yes | 126985 | 19256 | 785789.35 | 24.5053 | 1.213 (1.193, 1.233) |  |
| Sex | Male | No | 1373879 | 75669 | 9312074.03 | 8.1259 | 1 (Ref.) | 0.9888 |
|  |  | Yes | 122267 | 12619 | 772646.59 | 16.3322 | 1.311 (1.285, 1.336) |  |
|  | Female | No | 778957 | 49039 | 5363709.21 | 9.1427 | 1 (Ref.) |  |
|  |  | Yes | 172283 | 17711 | 1142712.19 | 15.4991 | 1.330 (1.307, 1.353) |  |
| Income | Q2-4 | No | 1703851 | 96961 | 11642377.13 | 8.3283 | 1 (Ref.) | 0.1467 |
|  |  | Yes | 230078 | 23719 | 1507069.83 | 15.7385 | 1.310 (1.291, 1.329) |  |
|  | Q1 | No | 448985 | 27747 | 3033406.12 | 9.1471 | 1 (Ref.) |  |
|  |  | Yes | 64472 | 6611 | 408288.95 | 16.192 | 1.363 (1.326, 1.401) |  |
| BMI | <25 | No | 1103075 | 66848 | 7458335.4 | 8.9629 | 1 (Ref.) | <.0001 |
|  |  | Yes | 158417 | 16314 | 1013283.91 | 16.1001 | 1.275 (1.253, 1.298) |  |
|  | 25+ | No | 1049761 | 57860 | 7217447.85 | 8.0167 | 1 (Ref.) |  |
|  |  | Yes | 136133 | 14016 | 902074.87 | 15.5375 | 1.375 (1.349, 1.401) |  |
| Smoking | Non, ex | No | 1550321 | 94119 | 10611864.27 | 8.8692 | 1 (Ref.) | 0.2944 |
|  |  | Yes | 245016 | 25481 | 1603939.92 | 15.8865 | 1.313 (1.294, 1.331) |  |
|  | Current | No | 602515 | 30589 | 4063918.97 | 7.527 | 1 (Ref.) |  |
|  |  | Yes | 49534 | 4849 | 311418.86 | 15.5707 | 1.365 (1.323, 1.408) |  |
| Drinking | Non, Mild | No | 1917949 | 113564 | 13080133.87 | 8.6822 | 1 (Ref.) | 0.8971 |
|  |  | Yes | 277906 | 28941 | 1808114.88 | 16.0062 | 1.327 (1.310, 1.345) |  |
|  | Heavy | No | 234887 | 11144 | 1595649.38 | 6.984 | 1 (Ref.) |  |
|  |  | Yes | 16644 | 1389 | 107243.9 | 12.9518 | 1.388 (1.312, 1.468) |  |
| Regular exercise | No | No | 1701708 | 100358 | 11553005.22 | 8.6867 | 1 (Ref.) | 0.3507 |
|  |  | Yes | 236098 | 25074 | 1523976.22 | 16.453 | 1.313 (1.294, 1.332) |  |
|  | Yes | No | 451128 | 24350 | 3122778.02 | 7.7975 | 1 (Ref.) |  |
|  |  | Yes | 58452 | 5256 | 391382.56 | 13.4293 | 1.361 (1.321, 1.403) |  |
| Hypertension | No | No | 987111 | 37064 | 6804108.35 | 5.4473 | 1 (Ref.) | <.0001 |
|  |  | Yes | 106080 | 7536 | 703872.04 | 10.7065 | 1.396 (1.362, 1.432) |  |
|  | Yes | No | 1165725 | 87644 | 7871674.9 | 11.1341 | 1 (Ref.) |  |
|  |  | Yes | 188470 | 22794 | 1211486.74 | 18.8149 | 1.297 (1.277, 1.316) |  |
| Dyslipidemia | No | No | 1295697 | 69109 | 8820652.94 | 7.8349 | 1 (Ref.) | 0.959 |
|  |  | Yes | 144799 | 13908 | 935186.26 | 14.8719 | 1.310 (1.285, 1.334) |  |
|  | Yes | No | 857139 | 55599 | 5855130.31 | 9.4958 | 1 (Ref.) |  |
|  |  | Yes | 149751 | 16422 | 980172.52 | 16.7542 | 1.331 (1.308, 1.355) |  |
| CKD | No | No | 1941064 | 99045 | 13283884.59 | 7.456 | 1 (Ref.) | <.0001 |
|  |  | Yes | 246929 | 22386 | 1624113.3 | 13.7835 | 1.371 (1.351, 1.392) |  |
|  | Yes | No | 211772 | 25663 | 1391898.66 | 18.4374 | 1 (Ref.) |  |
|  |  | Yes | 47621 | 7944 | 291245.48 | 27.276 | 1.188 (1.157, 1.219) |  |
| CVD History | No | No | 2116576 | 119497 | 14447471.57 | 8.2711 | 1 (Ref.) | 0.0018 |
|  |  | Yes | 283469 | 28234 | 1850207.83 | 15.2599 | 1.329 (1.311, 1.347) |  |
|  | Yes | No | 36260 | 5211 | 228311.68 | 22.8241 | 1 (Ref.) |  |
|  |  | Yes | 11081 | 2096 | 65150.95 | 32.1714 | 1.208 (1.146, 1.273) |  |
| Insulin use | No | No | 1996158 | 105535 | 13669036.2 | 7.7207 | 1 (Ref.) | 0.0661 |
|  |  | Yes | 251087 | 22837 | 1652275.86 | 13.8215 | 1.339 (1.320, 1.359) |  |
|  | Yes | No | 156678 | 19173 | 1006747.05 | 19.0445 | 1 (Ref.) |  |
|  |  | Yes | 43463 | 7493 | 263082.92 | 28.4815 | 1.261 (1.226, 1.296) |  |
| OHA Number | <3 | No | 1858447 | 99466 | 12656780.78 | 7.8587 | 1 (Ref.) | <.0001 |
|  |  | Yes | 240880 | 23375 | 1565904.71 | 14.9275 | 1.334 (1.315, 1.354) |  |
|  | 3+ | No | 294389 | 25242 | 2019002.47 | 12.5022 | 1 (Ref.) |  |
|  |  | Yes | 53670 | 6955 | 349454.07 | 19.9025 | 1.277 (1.243, 1.312) |  |
| DM Duration | <5 | No | 1531353 | 68672 | 10460509.1 | 6.5649 | 1 (Ref.) | 0.0076 |
|  |  | Yes | 175465 | 14505 | 1149355.54 | 12.6201 | 1.400 (1.374, 1.426) |  |
|  | 5+ | No | 621483 | 56036 | 4215274.15 | 13.2936 | 1 (Ref.) |  |
|  |  | Yes | 119085 | 15825 | 766003.24 | 20.6592 | 1.252 (1.229, 1.275) |  |

Competing risk model 5: Adjusted for age, sex, BMI, low income, smoking, drinking, regular exercise, hypertension, dyslipidemia, CKD, fasting glucose, duration of DM ≥5 years, insulin use, use of > 3 types of oral hypoglycemic agents, and cardiovascular diseases.

Abbreviations: Q, quartile; HR, hazard ratio; aHR, adjusted hazard ratio; CI, confidence interval; BMI, body mass index; DM, diabetes mellitus; CKD, chronic kidney disease; OHA, oral hypoglycemic agent.

**Supplemental Table S10. Subgroup analysis–Bipolar disorder**

|  |  | Bipolar | N | HF | Duration | Rate | Competeing risk Model 5 | * p for interaction |
| --- | --- | --- | --- | --- | --- | --- | --- | --- |
| Age groups | <40 | No | 195015 | 2295 | 1356563.34 | 1.6918 | 1 (Ref.) | **0.0296** |
|  |  | Yes | 369 | 9 | 2500.45 | 3.5993 | 2.033 (1.057, 3.911) |  |
|  | 40-64 | No | 1555978 | 65434 | 10761196.81 | 6.0806 | 1 (Ref.) |  |
|  |  | Yes | 4903 | 332 | 32347.37 | 10.2636 | 1.607 (1.443, 1.790) |  |
|  | ≥65 | No | 689109 | 86653 | 4426542.49 | 19.5758 | 1 (Ref.) |  |
|  |  | Yes | 2012 | 315 | 11991.56 | 26.2685 | 1.233 (1.102, 1.380) |  |
| Sex | Male | No | 1492614 | 87985 | 10062221.1 | 8.7441 | 1 (Ref.) | 0.6556 |
|  |  | Yes | 3532 | 303 | 22499.52 | 13.467 | 1.306 (1.165, 1.464) |  |
|  | Female | No | 947488 | 66397 | 6482081.53 | 10.2432 | 1 (Ref.) |  |
|  |  | Yes | 3752 | 353 | 24339.87 | 14.503 | 1.506 (1.354, 1.675) |  |
| Income | Q2-4 | No | 1928650 | 120176 | 13115046.75 | 9.1632 | 1 (Ref.) | 1 |
|  |  | Yes | 5279 | 504 | 34400.21 | 14.6511 | 1.407 (1.287, 1.537) |  |
|  | Q1 | No | 511452 | 34206 | 3429255.89 | 9.9748 | 1 (Ref.) |  |
|  |  | Yes | 2005 | 152 | 12439.18 | 12.2195 | 1.406 (1.197, 1.651) |  |
| BMI | <25 | No | 1258241 | 82852 | 8451022.35 | 9.8038 | 1 (Ref.) | 1 |
|  |  | Yes | 3251 | 310 | 20596.95 | 15.0508 | 1.381 (1.233, 1.547) |  |
|  | 25+ | No | 1181861 | 71530 | 8093280.28 | 8.8382 | 1 (Ref.) |  |
|  |  | Yes | 4033 | 346 | 26242.44 | 13.1848 | 1.435 (1.290, 1.597) |  |
| Smoking | Non, ex | No | 1790002 | 119093 | 12181438.25 | 9.7766 | 1 (Ref.) | 0.9996 |
|  |  | Yes | 5335 | 507 | 34365.95 | 14.753 | 1.431 (1.309, 1.564) |  |
|  | Current | No | 650100 | 35289 | 4362864.39 | 8.0885 | 1 (Ref.) |  |
|  |  | Yes | 1949 | 149 | 12473.44 | 11.9454 | 1.328 (1.131, 1.560) |  |
| Drinking | Non, Mild | No | 2188915 | 141874 | 14843589.12 | 9.5579 | 1 (Ref.) | 1 |
|  |  | Yes | 6940 | 631 | 44659.63 | 14.1291 | 1.433 (1.323, 1.551) |  |
|  | Heavy | No | 251187 | 12508 | 1700713.52 | 7.3546 | 1 (Ref.) |  |
|  |  | Yes | 344 | 25 | 2179.76 | 11.4692 | 1.362 (0.915, 2.028) |  |
| Regular exercise | No | No | 1931896 | 124868 | 13039379.14 | 9.5762 | 1 (Ref.) | 0.5787 |
|  |  | Yes | 5910 | 564 | 37602.31 | 14.9991 | 1.453 (1.336, 1.581) |  |
|  | Yes | No | 508206 | 29514 | 3504923.5 | 8.4207 | 1 (Ref.) |  |
|  |  | Yes | 1374 | 92 | 9237.08 | 9.9599 | 1.175 (0.958, 1.442) |  |
| Hypertension | No | No | 1089591 | 44367 | 7484331.87 | 5.928 | 1 (Ref.) | 0.9589 |
|  |  | Yes | 3600 | 233 | 23648.53 | 9.8526 | 1.507 (1.324, 1.715) |  |
|  | Yes | No | 1350511 | 110015 | 9059970.77 | 12.143 | 1 (Ref.) |  |
|  |  | Yes | 3684 | 423 | 23190.86 | 18.2399 | 1.357 (1.231, 1.495) |  |
| Dyslipidemia | No | No | 1436615 | 82682 | 9730868.23 | 8.4969 | 1 (Ref.) | 0.9433 |
|  |  | Yes | 3881 | 335 | 24970.97 | 13.4156 | 1.483 (1.330, 1.653) |  |
|  | Yes | No | 1003487 | 71700 | 6813434.41 | 10.5233 | 1 (Ref.) |  |
|  |  | Yes | 3403 | 321 | 21868.42 | 14.6787 | 1.335 (1.194, 1.492) |  |
| CKD | No | No | 2181605 | 120918 | 14866634.51 | 8.1335 | 1 (Ref.) | 0.4962 |
|  |  | Yes | 6388 | 513 | 41363.38 | 12.4023 | 1.471 (1.348, 1.605) |  |
|  | Yes | No | 258497 | 33464 | 1677668.13 | 19.9467 | 1 (Ref.) |  |
|  |  | Yes | 896 | 143 | 5476.01 | 26.1139 | 1.215 (1.028, 1.437) |  |
| CVD History | No | No | 2392985 | 147117 | 16252179.99 | 9.0521 | 1 (Ref.) | 0.5908 |
|  |  | Yes | 7060 | 614 | 45499.4 | 13.4947 | 1.427 (1.317, 1.546) |  |
|  | Yes | No | 47117 | 7265 | 292122.64 | 24.8697 | 1 (Ref.) |  |
|  |  | Yes | 224 | 42 | 1339.99 | 31.3436 | 1.161 (0.844, 1.598) |  |
| Insulin use | No | No | 2240867 | 127857 | 15279890.26 | 8.3677 | 1 (Ref.) | 0.9979 |
|  |  | Yes | 6378 | 515 | 41421.8 | 12.4331 | 1.467 (1.344, 1.600) |  |
|  | Yes | No | 199235 | 26525 | 1264412.38 | 20.9781 | 1 (Ref.) |  |
|  |  | Yes | 906 | 141 | 5417.59 | 26.0263 | 1.223 (1.032, 1.449) |  |
| OHA Number | <3 | No | 2093293 | 122326 | 14183755.23 | 8.6244 | 1 (Ref.) | 0.289 |
|  |  | Yes | 6034 | 515 | 38930.26 | 13.2288 | 1.435 (1.315, 1.566) |  |
|  | 3+ | No | 346809 | 32056 | 2360547.41 | 13.5799 | 1 (Ref.) |  |
|  |  | Yes | 1250 | 141 | 7909.13 | 17.8275 | 1.312 (1.108, 1.553) |  |
| DM Duration | <5 | No | 1701968 | 82817 | 11578295.55 | 7.1528 | 1 (Ref.) | 0.969 |
|  |  | Yes | 4850 | 360 | 31569.08 | 11.4036 | 1.531 (1.379, 1.699) |  |
|  | 5+ | No | 738134 | 71565 | 4966007.08 | 14.411 | 1 (Ref.) |  |
|  |  | Yes | 2434 | 296 | 15270.31 | 19.384 | 1.280 (1.140, 1.437) |  |


Competing risk model 5: Adjusted for age, sex, BMI, low income, smoking, drinking, regular exercise, hypertension, dyslipidemia, CKD, fasting glucose, duration of DM ≥5 years, insulin use, use of more than 3 types of oral hypoglycemic agents, and cardiovascular disease.

Abbreviations: Q, quartile; HR, hazard ratio; aHR, adjusted hazard ratio; CI, confidence interval; BMI, body mass index; DM, diabetes mellitus; CKD, chronic kidney disease; OHA, oral hypoglycemic agent.

**Supplemental Table S11. Subgroup analysis–Schizophrenia**

|  |  | Schizophrenia | N | HF | Duration | Rate | Competing risk Model 5 | * p for interaction |
| --- | --- | --- | --- | --- | --- | --- | --- | --- |
| Age groups | <40 | No | 194721 | 2283 | 1354587.02 | 1.6854 | 1 (Ref.) | <.0001 |
|  |  | Yes | 663 | 21 | 4476.78 | 4.6909 | 2.519 (1.638, 3.872) |  |
|  | 40-64 | No | 1552865 | 65308 | 10742307.38 | 6.0795 | 1 (Ref.) |  |
|  |  | Yes | 8016 | 458 | 51236.81 | 8.9389 | 1.593 (1.453, 1.747) |  |
|  | ≥65 | No | 689488 | 86755 | 4429085.1 | 19.5876 | 1 (Ref.) |  |
|  |  | Yes | 1633 | 213 | 9448.95 | 22.5422 | 1.082 (0.944, 1.240) |  |
| Sex | Male | No | 1490899 | 87939 | 10051807.52 | 8.7486 | 1 (Ref.) | 1 |
|  |  | Yes | 5247 | 349 | 32913.11 | 10.6037 | 1.393 (1.252, 1.549) |  |
|  | Female | No | 946175 | 66407 | 6474171.97 | 10.2572 | 1 (Ref.) |  |
|  |  | Yes | 5065 | 343 | 32249.43 | 10.6358 | 1.418 (1.272, 1.580) |  |
| Income | Q2-4 | No | 1927924 | 120256 | 13110056.73 | 9.1728 | 1 (Ref.) | 0.149 |
|  |  | Yes | 6005 | 424 | 39390.24 | 10.7641 | 1.306 (1.186, 1.439) |  |
|  | Q1 | No | 509150 | 34090 | 3415922.76 | 9.9797 | 1 (Ref.) |  |
|  |  | Yes | 4307 | 268 | 25772.3 | 10.3988 | 1.597 (1.413, 1.804) |  |
| BMI | <25 | No | 1256990 | 82861 | 8443849.56 | 9.8132 | 1 (Ref.) | 0.2893 |
|  |  | Yes | 4502 | 301 | 27769.75 | 10.8391 | 1.279 (1.140, 1.436) |  |
|  | 25+ | No | 1180084 | 71485 | 8082129.93 | 8.8448 | 1 (Ref.) |  |
|  |  | Yes | 5810 | 391 | 37392.79 | 10.4566 | 1.526 (1.380, 1.688) |  |
| Smoking | Non, ex | No | 1788140 | 119105 | 12170158.34 | 9.7866 | 1 (Ref.) | 1 |
|  |  | Yes | 7197 | 495 | 45645.86 | 10.8444 | 1.408 (1.287, 1.541) |  |
|  | Current | No | 648934 | 35241 | 4355821.15 | 8.0906 | 1 (Ref.) |  |
|  |  | Yes | 3115 | 197 | 19516.68 | 10.0939 | 1.394 (1.210, 1.607) |  |
| Drinking | Non, Mild | No | 2185898 | 141835 | 14825280.9 | 9.5671 | 1 (Ref.) | 1 |
|  |  | Yes | 9957 | 670 | 62967.85 | 10.6403 | 1.450 (1.342, 1.566) |  |
|  | Heavy | No | 251176 | 12511 | 1700698.59 | 7.3564 | 1 (Ref.) |  |
|  |  | Yes | 355 | 22 | 2194.68 | 10.0242 | 1.315 (0.860, 2.010) |  |
| Regular exercise | No | No | 1929030 | 124823 | 13021945.56 | 9.5856 | 1 (Ref.) | 0.9992 |
|  |  | Yes | 8776 | 609 | 55035.89 | 11.0655 | 1.423 (1.312, 1.543) |  |
|  | Yes | No | 508044 | 29523 | 3504033.93 | 8.4254 | 1 (Ref.) |  |
|  |  | Yes | 1536 | 83 | 10126.65 | 8.1962 | 1.288 (1.036, 1.600) |  |
| Hypertension | No | No | 1087253 | 44309 | 7469612.73 | 5.9319 | 1 (Ref.) | 1 |
|  |  | Yes | 5938 | 291 | 38367.66 | 7.5845 | 1.427 (1.270, 1.602) |  |
|  | Yes | No | 1349821 | 110037 | 9056366.76 | 12.1502 | 1 (Ref.) |  |
|  |  | Yes | 4374 | 401 | 26794.88 | 14.9655 | 1.390 (1.257, 1.537) |  |
| Dyslipidemia | No | No | 1434522 | 82634 | 9718195.12 | 8.503 | 1 (Ref.) | 0.9988 |
|  |  | Yes | 5974 | 383 | 37644.08 | 10.1742 | 1.449 (1.309, 1.605) |  |
|  | Yes | No | 1002552 | 71712 | 6807784.37 | 10.5338 | 1 (Ref.) |  |
|  |  | Yes | 4338 | 309 | 27518.46 | 11.2288 | 1.354 (1.208, 1.517) |  |
| CKD | No | No | 2178762 | 120866 | 14849374.43 | 8.1395 | 1 (Ref.) | 0.1837 |
|  |  | Yes | 9231 | 565 | 58623.46 | 9.6378 | 1.477 (1.358, 1.606) |  |
|  | Yes | No | 258312 | 33480 | 1676605.06 | 19.9689 | 1 (Ref.) |  |
|  |  | Yes | 1081 | 127 | 6539.08 | 19.4217 | 1.155 (0.967, 1.380) |  |
| CVD History | No | No | 2389949 | 147067 | 16233741.9 | 9.0593 | 1 (Ref.) | 1 |
|  |  | Yes | 10096 | 664 | 63937.5 | 10.3851 | 1.422 (1.316, 1.537) |  |
|  | Yes | No | 47125 | 7279 | 292237.59 | 24.9078 | 1 (Ref.) |  |
|  |  | Yes | 216 | 28 | 1225.04 | 22.8564 | 1.094 (0.749, 1.598) |  |
| Insulin use | No | No | 2237936 | 127809 | 15261830.83 | 8.3744 | 1 (Ref.) | 1 |
|  |  | Yes | 9309 | 563 | 59481.23 | 9.4652 | 1.422 (1.308, 1.546) |  |
|  | Yes | No | 199138 | 26537 | 1264148.66 | 20.992 | 1 (Ref.) |  |
|  |  | Yes | 1003 | 129 | 5681.31 | 22.706 | 1.336 (1.115, 1.601) |  |
| OHA Number | <3 | No | 2090675 | 122295 | 14167800.36 | 8.6319 | 1 (Ref.) | 1 |
|  |  | Yes | 8652 | 546 | 54885.13 | 9.9481 | 1.400 (1.286, 1.525) |  |
|  | 3+ | No | 346399 | 32051 | 2358179.13 | 13.5914 | 1 (Ref.) |  |
|  |  | Yes | 1660 | 146 | 10277.41 | 14.2059 | 1.423 (1.206, 1.678) |  |
| DM Duration | <5 | No | 1699411 | 82776 | 11562311.85 | 7.1591 | 1 (Ref.) | 0.9417 |
|  |  | Yes | 7407 | 401 | 47552.78 | 8.4327 | 1.392 (1.260, 1.538) |  |
|  | 5+ | No | 737663 | 71570 | 4963667.64 | 14.4188 | 1 (Ref.) |  |
|  |  | Yes | 2905 | 291 | 17609.75 | 16.5249 | 1.423 (1.265, 1.601) |  |

Competing risk model 5: Adjusted for age, sex, BMI, low income, smoking, drinking, regular exercise, hypertension, dyslipidemia, CKD, fasting glucose, duration of DM ≥5 years, insulin use, use of more than 3 types of oral hypoglycemic agents, and cardiovascular disease.

Abbreviations: Q, quartile; HR, hazard ratio; aHR, adjusted hazard ratio; CI, confidence interval; BMI, body mass index; DM, diabetes mellitus; CKD, chronic kidney disease; OHA, oral hypoglycemic agent.

**Supplemental Table S12. Subgroup analysis–Insomnia**

|  |  | Insomnia | N | HF | Duration | Rate | Competing risk Model 5 | * p for interaction |
| --- | --- | --- | --- | --- | --- | --- | --- | --- |
| Age groups | <40 | No | 188695 | 2151 | 1313689.89 | 1.6374 | 1 (Ref.) | <.0001 |
|  |  | Yes | 6689 | 153 | 45373.91 | 3.372 | 2.042 (1.734, 2.404) |  |
|  | 40-64 | No | 1398209 | 55037 | 9699309.16 | 5.6743 | 1 (Ref.) |  |
|  |  | Yes | 162672 | 10729 | 1094235.02 | 9.805 | 1.497 (1.466, 1.528) |  |
|  | ≥65 | No | 550052 | 65729 | 3567227.98 | 18.4258 | 1 (Ref.) |  |
|  |  | Yes | 141069 | 21239 | 871306.07 | 24.376 | 1.211 (1.192, 1.231) |  |
| Sex | Male | No | 1366899 | 74702 | 9274396.78 | 8.0546 | 1 (Ref.) | 1 |
|  |  | Yes | 129247 | 13586 | 810323.85 | 16.7661 | 1.311 (1.286, 1.336) |  |
|  | Female | No | 770057 | 48215 | 5305830.25 | 9.0872 | 1 (Ref.) |  |
|  |  | Yes | 181183 | 18535 | 1200591.15 | 15.4382 | 1.301 (1.278, 1.323) |  |
| Income | Q2-4 | No | 1693430 | 95823 | 11581154.85 | 8.274 | 1 (Ref.) | 0.0276 |
|  |  | Yes | 240499 | 24857 | 1568292.12 | 15.8497 | 1.292 (1.273, 1.310) |  |
|  | Q1 | No | 443526 | 27094 | 2999072.18 | 9.0341 | 1 (Ref.) |  |
|  |  | Yes | 69931 | 7264 | 442622.88 | 16.4113 | 1.354 (1.318, 1.390) |  |
| BMI | <25 | No | 1091571 | 65560 | 7390640.37 | 8.8707 | 1 (Ref.) | <.0001 |
|  |  | Yes | 169921 | 17602 | 1080978.93 | 16.2834 | 1.263 (1.242, 1.285) |  |
|  | 25+ | No | 1045385 | 57357 | 7189586.66 | 7.9778 | 1 (Ref.) |  |
|  |  | Yes | 140509 | 14519 | 929936.06 | 15.6129 | 1.355 (1.330, 1.381) |  |
| Smoking | Non, ex | No | 1539361 | 92879 | 10545320.85 | 8.8076 | 1 (Ref.) | 0.0231 |
|  |  | Yes | 255976 | 26721 | 1670483.35 | 15.996 | 1.294 (1.276, 1.312) |  |
|  | Current | No | 597595 | 30038 | 4034906.18 | 7.4445 | 1 (Ref.) |  |
|  |  | Yes | 54454 | 5400 | 340431.65 | 15.8622 | 1.363 (1.323, 1.404) |  |
| Drinking | Non, Mild | No | 1904364 | 111993 | 12998453.62 | 8.6159 | 1 (Ref.) | 0.1953 |
|  |  | Yes | 291491 | 30512 | 1889795.13 | 16.1457 | 1.307 (1.290, 1.325) |  |
|  | Heavy | No | 232592 | 10924 | 1581773.41 | 6.9062 | 1 (Ref.) |  |
|  |  | Yes | 18939 | 1609 | 121119.87 | 13.2844 | 1.398 (1.326, 1.474) |  |
| Regular exercise | No | No | 1690280 | 98949 | 11485849.14 | 8.6149 | 1 (Ref.) | 0.6387 |
|  |  | Yes | 247526 | 26483 | 1591132.31 | 16.6441 | 1.298 (1.280, 1.317) |  |
|  | Yes | No | 446676 | 23968 | 3094377.89 | 7.7457 | 1 (Ref.) |  |
|  |  | Yes | 62904 | 5638 | 419782.69 | 13.4308 | 1.337 (1.299, 1.377) |  |
| Hypertension | No | No | 984085 | 36692 | 6787470.01 | 5.4058 | 1 (Ref.) | <.0001 |
|  |  | Yes | 109106 | 7908 | 720510.38 | 10.9756 | 1.394 (1.360, 1.429) |  |
|  | Yes | No | 1152871 | 86225 | 7792757.02 | 11.0648 | 1 (Ref.) |  |
|  |  | Yes | 201324 | 24213 | 1290404.61 | 18.7639 | 1.277 (1.259, 1.296) |  |
| Dyslipidemia | No | No | 1282530 | 67506 | 8740939.69 | 7.723 | 1 (Ref.) | 1 |
|  |  | Yes | 157966 | 15511 | 1014899.51 | 15.2833 | 1.305 (1.282, 1.328) |  |
|  | Yes | No | 854426 | 55411 | 5839287.34 | 9.4893 | 1 (Ref.) |  |
|  |  | Yes | 152464 | 16610 | 996015.49 | 16.6764 | 1.306 (1.283, 1.329) |  |
| CKD | No | No | 1926843 | 97568 | 13197759.65 | 7.3928 | 1 (Ref.) | <.0001 |
|  |  | Yes | 261150 | 23863 | 1710238.24 | 13.953 | 1.350 (1.331, 1.370) |  |
|  | Yes | No | 210113 | 25349 | 1382467.38 | 18.3361 | 1 (Ref.) |  |
|  |  | Yes | 49280 | 8258 | 300676.76 | 27.4647 | 1.182 (1.153, 1.213) |  |
| CVD History | No | No | 2100365 | 117632 | 14349407.74 | 8.1977 | 1 (Ref.) | 0.001 |
|  |  | Yes | 299680 | 30099 | 1948271.66 | 15.4491 | 1.316 (1.299, 1.334) |  |
|  | Yes | No | 36591 | 5285 | 230819.29 | 22.8967 | 1 (Ref.) |  |
|  |  | Yes | 10750 | 2022 | 62643.34 | 32.278 | 1.144 (1.085, 1.206) |  |
| Insulin use | No | No | 1978808 | 103504 | 13562047.37 | 7.6319 | 1 (Ref.) | <.0001 |
|  |  | Yes | 268437 | 24868 | 1759264.68 | 14.1355 | 1.323 (1.304, 1.342) |  |
|  | Yes | No | 158148 | 19413 | 1018179.66 | 19.0664 | 1 (Ref.) |  |
|  |  | Yes | 41993 | 7253 | 251650.32 | 28.8217 | 1.242 (1.208, 1.277) |  |
| OHA Number | <3 | No | 1844317 | 97812 | 12571771.58 | 7.7803 | 1 (Ref.) | <.0001 |
|  |  | Yes | 255010 | 25029 | 1650913.9 | 15.1607 | 1.329 (1.310, 1.348) |  |
|  | 3+ | No | 292639 | 25105 | 2008455.45 | 12.4997 | 1 (Ref.) |  |
|  |  | Yes | 55420 | 7092 | 360001.09 | 19.6999 | 1.227 (1.194, 1.260) |  |
| DM Duration | <5 | No | 1518086 | 67241 | 10379028.51 | 6.4785 | 1 (Ref.) | <.0001 |
|  |  | Yes | 188732 | 15936 | 1230836.12 | 12.9473 | 1.382 (1.358, 1.407) |  |
|  | 5+ | No | 618870 | 55676 | 4201198.52 | 13.2524 | 1 (Ref.) |  |
|  |  | Yes | 121698 | 16185 | 780078.88 | 20.7479 | 1.234 (1.212, 1.257) |  |

Competing risk model 5: Adjusted for age, sex, BMI, low income, smoking, drinking, regular exercise, hypertension, dyslipidemia, CKD, fasting glucose, duration of DM ≥5 years, insulin use, use of more than 3 types of oral hypoglycemic agents, and cardiovascular disease.

Abbreviations: Q, quartile; HR, hazard ratio; aHR, adjusted hazard ratio; CI, confidence interval; BMI, body mass index; DM, diabetes mellitus; CKD, chronic kidney disease; OHA, oral hypoglycemic agent.

**Supplemental Table S 13. Subgroup analysis­–Anxiety disorders**

|  |  | Anxiety | N | HF | Duration | Rate | Competing risk Model 5 | * p for interaction |
| --- | --- | --- | --- | --- | --- | --- | --- | --- |
| Age groups | <40 | No | 180981 | 2066 | 1260502.42 | 1.639 | 1 (Ref.) | <.0001 |
|  |  | Yes | 14403 | 238 | 98561.38 | 2.4147 | 1.470 (1.286, 1.681) |  |
|  | 40-64 | No | 1263471 | 48381 | 8760719.26 | 5.5225 | 1 (Ref.) |  |
|  |  | Yes | 297410 | 17385 | 2032824.92 | 8.5521 | 1.396 (1.372, 1.421) |  |
|  | ≥65 | No | 469808 | 54973 | 3041225.53 | 18.0759 | 1 (Ref.) |  |
|  |  | Yes | 221313 | 31995 | 1397308.52 | 22.8976 | 1.222 (1.205, 1.240) |  |
| Sex | Male | No | 1269390 | 67321 | 8616119.43 | 7.8134 | 1 (Ref.) | 0.9813 |
|  |  | Yes | 226756 | 20967 | 1468601.2 | 14.2769 | 1.279 (1.259, 1.299) |  |
|  | Female | No | 644870 | 38099 | 4446327.79 | 8.5686 | 1 (Ref.) |  |
|  |  | Yes | 306370 | 28651 | 2060093.61 | 13.9076 | 1.296 (1.276, 1.316) |  |
| Income | Q2-4 | No | 1515982 | 81923 | 10368604.97 | 7.9011 | 1 (Ref.) | 0.683 |
|  |  | Yes | 417947 | 38757 | 2780841.99 | 13.9371 | 1.281 (1.265, 1.297) |  |
|  | Q1 | No | 398278 | 23497 | 2693842.25 | 8.7225 | 1 (Ref.) |  |
|  |  | Yes | 115179 | 10861 | 747852.81 | 14.5229 | 1.311 (1.281, 1.342) |  |
| BMI | <25 | No | 979212 | 56842 | 6627405.32 | 8.5768 | 1 (Ref.) | <.0001 |
|  |  | Yes | 282280 | 26320 | 1844213.99 | 14.2717 | 1.238 (1.220, 1.257) |  |
|  | 25+ | No | 935048 | 48578 | 6435041.9 | 7.549 | 1 (Ref.) |  |
|  |  | Yes | 250846 | 23298 | 1684480.82 | 13.831 | 1.346 (1.324, 1.367) |  |
| Smoking | Non, ex | No | 1351399 | 77877 | 9259469.06 | 8.4105 | 1 (Ref.) | 0.8213 |
|  |  | Yes | 443938 | 41723 | 2956335.14 | 14.1131 | 1.282 (1.266, 1.298) |  |
|  | Current | No | 562861 | 27543 | 3802978.16 | 7.2425 | 1 (Ref.) |  |
|  |  | Yes | 89188 | 7895 | 572359.67 | 13.7938 | 1.311 (1.278, 1.345) |  |
| Drinking | Non, Mild | No | 1695747 | 95336 | 11576769.82 | 8.2351 | 1 (Ref.) | 0.998 |
|  |  | Yes | 500108 | 47169 | 3311478.93 | 14.2441 | 1.293 (1.279, 1.308) |  |
|  | Heavy | No | 218513 | 10084 | 1485677.4 | 6.7875 | 1 (Ref.) |  |
|  |  | Yes | 33018 | 2449 | 217215.88 | 11.2745 | 1.324 (1.266, 1.384) |  |
| Regular exercise | No | No | 1512619 | 84614 | 10280315.13 | 8.2307 | 1 (Ref.) | 0.8676 |
|  |  | Yes | 425187 | 40818 | 2796666.32 | 14.5952 | 1.282 (1.267, 1.298) |  |
|  | Yes | No | 401641 | 20806 | 2782132.08 | 7.4784 | 1 (Ref.) |  |
|  |  | Yes | 107939 | 8800 | 732028.49 | 12.0214 | 1.310 (1.277, 1.343) |  |
| Hypertension | No | No | 897343 | 32274 | 6187052.35 | 5.2164 | 1 (Ref.) | 0.0821 |
|  |  | Yes | 195848 | 12326 | 1320928.05 | 9.3313 | 1.320 (1.293, 1.348) |  |
|  | Yes | No | 1016917 | 73146 | 6875394.87 | 10.6388 | 1 (Ref.) |  |
|  |  | Yes | 337278 | 37292 | 2207766.76 | 16.8913 | 1.276 (1.260, 1.293) |  |
| Dyslipidemia | No | No | 1167368 | 59272 | 7955067.41 | 7.4508 | 1 (Ref.) | 1 |
|  |  | Yes | 273128 | 23745 | 1800771.79 | 13.186 | 1.283 (1.263, 1.303) |  |
|  | Yes | No | 746892 | 46148 | 5107379.81 | 9.0356 | 1 (Ref.) |  |
|  |  | Yes | 259998 | 25873 | 1727923.02 | 14.9735 | 1.292 (1.272, 1.312) |  |
| CKD | No | No | 1732870 | 84141 | 11869290.57 | 7.089 | 1 (Ref.) | <.0001 |
|  |  | Yes | 455123 | 37290 | 3038707.32 | 12.2717 | 1.323 (1.306, 1.340) |  |
|  | Yes | No | 181390 | 21279 | 1193156.65 | 17.8342 | 1 (Ref.) |  |
|  |  | Yes | 78003 | 12328 | 489987.49 | 25.1598 | 1.180 (1.154, 1.207) |  |
| CVD History | No | No | 1883920 | 101104 | 12871603.58 | 7.8548 | 1 (Ref.) | <.0001 |
|  |  | Yes | 516125 | 46627 | 3426075.81 | 13.6094 | 1.296 (1.281, 1.311) |  |
|  | Yes | No | 30340 | 4316 | 190843.64 | 22.6154 | 1 (Ref.) |  |
|  |  | Yes | 17001 | 2991 | 102618.99 | 29.1467 | 1.144 (1.091, 1.201) |  |
| Insulin use | No | No | 1777599 | 89099 | 12184431.78 | 7.3125 | 1 (Ref.) | <.0001 |
|  |  | Yes | 469646 | 39273 | 3136880.28 | 12.5198 | 1.309 (1.293, 1.325) |  |
|  | Yes | No | 136661 | 16321 | 878015.44 | 18.5885 | 1 (Ref.) |  |
|  |  | Yes | 63480 | 10345 | 391814.53 | 26.4028 | 1.201 (1.171, 1.232) |  |
| OHA Number | <3 | No | 1656653 | 84023 | 11294487.01 | 7.4393 | 1 (Ref.) | <.0001 |
|  |  | Yes | 442674 | 38818 | 2928198.47 | 13.2566 | 1.306 (1.290, 1.323) |  |
|  | 3+ | No | 257607 | 21397 | 1767960.21 | 12.1026 | 1 (Ref.) |  |
|  |  | Yes | 90452 | 10800 | 600496.33 | 17.9851 | 1.221 (1.192, 1.250) |  |
| DM Duration | <5 | No | 1369804 | 57759 | 9368607.2 | 6.1652 | 1 (Ref.) | <.0001 |
|  |  | Yes | 337014 | 25418 | 2241257.43 | 11.341 | 1.365 (1.344, 1.386) |  |
|  | 5+ | No | 544456 | 47661 | 3693840.02 | 12.9028 | 1 (Ref.) |  |
|  |  | Yes | 196112 | 24200 | 1287437.38 | 18.797 | 1.210 (1.191, 1.229) |  |

Competing risk model 5: Adjusted for age, sex, BMI, low income, smoking, drinking, regular exercise, hypertension, dyslipidemia, CKD, fasting glucose, duration of DM ≥5 years, insulin use, use of more than 3 types of oral hypoglycemic agents, and cardiovascular disease.

Abbreviations: Q, quartile; HR, hazard ratio; aHR, adjusted hazard ratio; CI, confidence interval; BMI, body mass index; DM, diabetes mellitus; CKD, chronic kidney disease; OHA, oral hypoglycemic agent.

**Supplemental Table S14. Subgroup analysis according to age (20-39 years)**

|  |  | Mental disorder | Age 20-39 years | | | | | |
| --- | --- | --- | --- | --- | --- | --- | --- | --- |
|  |  |  | N | Event | Duration | IR | HR (95% C.I) | *P for interaction |
| Sex | Male | 0 | 143409 | 1590 | 1001351.76 | 1.58785 | 1 (Ref.) | 1 |
|  |  | 1 | 13274 | 189 | 91278.24 | 2.07059 | 1.206 (1.035, 1.404) |  |
|  |  | ≥2 | 2984 | 86 | 20201.11 | 4.25719 | 2.193 (1.754, 2.741) |  |
|  | Female | 0 | 28901 | 318 | 200046.23 | 1.58963 | 1 (Ref.) |  |
|  |  | 1 | 5130 | 70 | 34906.74 | 2.00534 | 1.178 (0.911, 1.525) |  |
|  |  | ≥2 | 1686 | 51 | 11279.72 | 4.52139 | 2.369 (1.761, 3.187) |  |
| Income | Q2-4 | 0 | 142278 | 1520 | 993454.21 | 1.53002 | 1 (Ref.) | 1 |
|  |  | 1 | 14566 | 193 | 100169.6 | 1.92673 | 1.197 (1.029, 1.391) |  |
|  |  | ≥2 | 3409 | 93 | 23194.08 | 4.00964 | 2.301 (1.860, 2.848) |  |
|  | Q1 | 0 | 30032 | 388 | 207943.78 | 1.86589 | 1 (Ref.) |  |
|  |  | 1 | 3838 | 66 | 26015.38 | 2.53696 | 1.199 (0.922, 1.559) |  |
|  |  | ≥2 | 1261 | 44 | 8286.76 | 5.30968 | 2.155 (1.565, 2.967) |  |
| Obesity | No | 0 | 77316 | 709 | 541589.82 | 1.30911 | 1 (Ref.) | 0.162 |
|  |  | 1 | 8451 | 114 | 58158.81 | 1.96015 | 1.366 (1.119, 1.667) |  |
|  |  | ≥2 | 2301 | 75 | 15509.66 | 4.8357 | 2.877 (2.249, 3.682) |  |
|  | Yes | 0 | 94994 | 1199 | 659808.16 | 1.81719 | 1 (Ref.) |  |
|  |  | 1 | 9953 | 145 | 68026.17 | 2.13153 | 1.097 (0.923, 1.304) |  |
|  |  | ≥2 | 2369 | 62 | 15971.17 | 3.88199 | 1.796 (1.385, 2.328) |  |
| Smoking | Non, Ex | 0 | 80803 | 803 | 564031.72 | 1.42368 | 1 (Ref.) | 0.691 |
|  |  | 1 | 9975 | 125 | 68500.95 | 1.82479 | 1.186 (0.981, 1.433) |  |
|  |  | ≥2 | 2698 | 62 | 18368.4 | 3.37536 | 1.841 (1.417, 2.393) |  |
|  | Current | 0 | 91507 | 1105 | 637366.27 | 1.7337 | 1 (Ref.) |  |
|  |  | 1 | 8429 | 134 | 57684.03 | 2.323 | 1.208 (1.008, 1.446) |  |
|  |  | ≥2 | 1972 | 75 | 13112.44 | 5.71976 | 2.752 (2.167, 3.495) |  |
| Drinking | Non, Mild | 0 | 147032 | 1580 | 1024899.16 | 1.54162 | 1 (Ref.) | 1 |
|  |  | 1 | 15938 | 216 | 109261.03 | 1.97692 | 1.185 (1.027, 1.368) |  |
|  |  | ≥2 | 4130 | 119 | 27878.9 | 4.26846 | 2.268 (1.873, 2.746) |  |
|  | Heavy | 0 | 25278 | 328 | 176498.82 | 1.85837 | 1 (Ref.) |  |
|  |  | 1 | 2466 | 43 | 16923.95 | 2.54078 | 1.282 (0.930, 1.768) |  |
|  |  | ≥2 | 540 | 18 | 3601.94 | 4.99731 | 2.272 (1.402, 3.681) |  |
| Regular exercise | No | 0 | 147824 | 1622 | 1029846.64 | 1.57499 | 1 (Ref.) | 0.6284 |
|  |  | 1 | 15663 | 221 | 107294.31 | 2.05976 | 1.221 (1.059, 1.407) |  |
|  |  | ≥2 | 3881 | 122 | 26094.19 | 4.67537 | 2.472 (2.050, 2.980) |  |
|  | Yes | 0 | 24486 | 286 | 171551.35 | 1.66714 | 1 (Ref.) |  |
|  |  | 1 | 2741 | 38 | 18890.67 | 2.01157 | 1.077 (0.766, 1.514) |  |
|  |  | ≥2 | 789 | 15 | 5386.64 | 2.78467 | 1.298 (0.756, 2.230) |  |
| Hypertension | No | 0 | 130819 | 1173 | 911902.25 | 1.28632 | 1 (Ref.) | 1 |
|  |  | 1 | 13818 | 148 | 94657.11 | 1.56354 | 1.153 (0.971, 1.370) |  |
|  |  | ≥2 | 3500 | 83 | 23621.59 | 3.51373 | 2.278 (1.810, 2.866) |  |
|  | Yes | 0 | 41491 | 735 | 289495.73 | 2.5389 | 1 (Ref.) |  |
|  |  | 1 | 4586 | 111 | 31527.87 | 3.5207 | 1.263 (1.033, 1.544) |  |
|  |  | ≥2 | 1170 | 54 | 7859.24 | 6.87089 | 2.219 (1.680, 2.931) |  |
| Dyslipidemia | No | 0 | 131135 | 1187 | 913380.82 | 1.29957 | 1 (Ref.) | 0.8833 |
|  |  | 1 | 13630 | 154 | 93325.17 | 1.65014 | 1.229 (1.038, 1.456) |  |
|  |  | ≥2 | 3294 | 85 | 22161.42 | 3.83549 | 2.609 (2.090, 3.259) |  |
|  | Yes | 0 | 41175 | 721 | 288017.16 | 2.50332 | 1 (Ref.) |  |
|  |  | 1 | 4774 | 105 | 32859.81 | 3.19539 | 1.150 (0.936, 1.414) |  |
|  |  | ≥2 | 1376 | 52 | 9319.41 | 5.57975 | 1.832 (1.372, 2.445) |  |
| CKD | No | 0 | 167588 | 1820 | 1165992.69 | 1.5609 | 1 (Ref.) | 0.9417 |
|  |  | 1 | 17887 | 240 | 122426.65 | 1.96036 | 1.175 (1.026, 1.346) |  |
|  |  | ≥2 | 4519 | 132 | 30408.47 | 4.3409 | 2.320 (1.934, 2.782) |  |
|  | Yes | 0 | 4722 | 88 | 35405.3 | 2.4855 | 1 (Ref.) |  |
|  |  | 1 | 517 | 19 | 3758.33 | 5.05543 | 1.583 (0.952, 2.633) |  |
|  |  | ≥2 | 151 | 5 | 1072.37 | 4.66258 | 1.290 (0.536, 3.102) |  |
| Insulin | No | 0 | 166459 | 1688 | 1160167.87 | 1.455 | 1 (Ref.) | 1 |
|  |  | 1 | 17386 | 209 | 119218.68 | 1.7531 | 1.186 (1.026, 1.371) |  |
|  |  | ≥2 | 4273 | 99 | 28863.26 | 3.43 | 2.248 (1.831, 2.760) |  |
|  | Yes | 0 | 5851 | 220 | 41230.12 | 5.3359 | 1 (Ref.) |  |
|  |  | 1 | 1018 | 50 | 6966.3 | 7.1774 | 1.252 (0.919, 1.706) |  |
|  |  | ≥2 | 397 | 38 | 2617.57 | 14.5173 | 2.284 (1.600, 3.262) |  |
| OHA | <3 | 0 | 165616 | 1740 | 1152947.56 | 1.50918 | 1 (Ref.) | 0.9441 |
|  |  | 1 | 17390 | 235 | 119071.53 | 1.9736 | 1.240 (1.081, 1.422) |  |
|  |  | ≥2 | 4358 | 122 | 29376.1 | 4.15304 | 2.338 (1.936, 2.823) |  |
|  | ≥3 | 0 | 6694 | 168 | 48450.43 | 3.46746 | 1 (Ref.) |  |
|  |  | 1 | 1014 | 24 | 7113.45 | 3.37389 | 0.880 (0.573, 1.350) |  |
|  |  | ≥2 | 312 | 15 | 2104.74 | 7.12678 | 1.697 (1.002, 2.874) |  |
| DM Duration | <5 years | 0 | 163887 | 1608 | 1141619.97 | 1.4085 | 1 (Ref.) | 1 |
|  |  | 1 | 16988 | 202 | 116367.3 | 1.7359 | 1.227 (1.059, 1.422) |  |
|  |  | ≥2 | 4208 | 99 | 28394.28 | 3.4866 | 2.305 (1.874, 2.834) |  |
|  | ≥5 years | 0 | 8423 | 300 | 59778.02 | 5.0186 | 1 (Ref.) |  |
|  |  | 1 | 1416 | 57 | 9817.68 | 5.8059 | 1.095 (0.824, 1.455) |  |
|  |  | ≥2 | 462 | 38 | 3086.55 | 12.3115 | 2.108 (1.494, 2.975) |  |

Competing risk model 5: Adjusted for age, sex, BMI, low income, smoking, drinking, regular exercise, hypertension, dyslipidemia, CKD, fasting glucose, duration of DM ≥5 years, insulin use, use of more than 3 types of oral hypoglycemic agents, and cardiovascular disease.

*Multiple testing was adjusted with Sidak method

Abbreviations: Q, quartile; IR, incidence rate; HR, hazard ratio; aHR, adjusted hazard ratio; CI, confidence interval; BMI, body mass index; DM, diabetes mellitus; CKD, chronic kidney disease; OHA, oral hypoglycemic agent.

**Supplemental Table S15. Subgroup analysis according to age (40-64 years)**

|  |  | Mental disorder | Age 40-65 years | | | | | |
| --- | --- | --- | --- | --- | --- | --- | --- | --- |
|  |  |  | N | Event | Duration | IR | Competing risk Model 5  HR (95% C.I) | *P for interaction |
| Sex | Male | 0 | 790718 | 30320 | 5466851.33 | 5.5462 | 1 (Ref.) | 0.0488 |
|  |  | 1 | 153762 | 8703 | 1042207.35 | 8.3505 | 1.293 (1.262, 1.324) |  |
|  |  | ≥2 | 59689 | 4811 | 387149.62 | 12.4267 | 1.675 (1.624, 1.728) |  |
|  | Female | 0 | 326026 | 9905 | 2295354.81 | 4.3152 | 1 (Ref.) |  |
|  |  | 1 | 144017 | 6289 | 1008477.58 | 6.2361 | 1.272 (1.232, 1.313) |  |
|  |  | ≥2 | 86669 | 5738 | 593503.5 | 9.668 | 1.785 (1.728, 1.845) |  |
| Income | Q2-4 | 0 | 880659 | 30249 | 6134598.23 | 4.9309 | 1 (Ref.) | 0.7822 |
|  |  | 1 | 229085 | 11192 | 1586758.43 | 7.0534 | 1.288 (1.260, 1.317) |  |
|  |  | ≥2 | 108334 | 7508 | 736954.98 | 10.1879 | 1.708 (1.664, 1.753) |  |
|  | Q1 | 0 | 236085 | 9976 | 1627607.9 | 6.1292 | 1 (Ref.) |  |
|  |  | 1 | 68694 | 3800 | 463926.5 | 8.191 | 1.266 (1.219, 1.315) |  |
|  |  | ≥2 | 38024 | 3041 | 243698.13 | 12.4786 | 1.786 (1.714, 1.862) |  |
| Obesity | No | 0 | 553977 | 20608 | 3844521.76 | 5.3604 | 1 (Ref.) | <.0001 |
|  |  | 1 | 149148 | 7482 | 1023928.88 | 7.3071 | 1.246 (1.214, 1.280) |  |
|  |  | ≥2 | 74909 | 5224 | 498544.22 | 10.4785 | 1.635 (1.585, 1.687) |  |
|  | Yes | 0 | 562767 | 19617 | 3917684.38 | 5.0073 | 1 (Ref.) |  |
|  |  | 1 | 148631 | 7510 | 1026756.05 | 7.3143 | 1.320 (1.285, 1.356) |  |
|  |  | ≥2 | 71449 | 5325 | 482108.89 | 11.0452 | 1.831 (1.775, 1.889) |  |
| Smoking | Non, Ex | 0 | 752484 | 25038 | 5273056.9 | 4.7483 | 1 (Ref.) | 1 |
|  |  | 1 | 228621 | 10813 | 1589811.4 | 6.8014 | 1.290 (1.260, 1.320) |  |
|  |  | ≥2 | 115907 | 7864 | 788363.72 | 9.9751 | 1.734 (1.689, 1.780) |  |
|  | Current | 0 | 364260 | 15187 | 2489149.24 | 6.1013 | 1 (Ref.) |  |
|  |  | 1 | 69158 | 4179 | 460873.53 | 9.0676 | 1.266 (1.224, 1.311) |  |
|  |  | ≥2 | 30451 | 2685 | 192289.4 | 13.9633 | 1.725 (1.654, 1.799) |  |
| Drinking | Non, Mild | 0 | 965806 | 34502 | 6726119.36 | 5.1296 | 1 (Ref.) | 1 |
|  |  | 1 | 269199 | 13487 | 1858241.86 | 7.2579 | 1.294 (1.268, 1.320) |  |
|  |  | ≥2 | 136182 | 9756 | 915143.47 | 10.6606 | 1.753 (1.712, 1.795) |  |
|  | Heavy | 0 | 150938 | 5723 | 1036086.78 | 5.5237 | 1 (Ref.) |  |
|  |  | 1 | 28580 | 1505 | 192443.07 | 7.8205 | 1.267 (1.196, 1.341) |  |
|  |  | ≥2 | 10176 | 793 | 65509.65 | 12.1051 | 1.754 (1.627, 1.890) |  |
| Regular exercise | No | 0 | 875775 | 31860 | 6067506.76 | 5.2509 | 1 (Ref.) | 1 |
|  |  | 1 | 232976 | 11878 | 1599389.97 | 7.4266 | 1.279 (1.252, 1.307) |  |
|  |  | ≥2 | 114513 | 8439 | 764107.78 | 11.0443 | 1.737 (1.694, 1.781) |  |
|  | Yes | 0 | 240969 | 8365 | 1694699.38 | 4.936 | 1 (Ref.) |  |
|  |  | 1 | 64803 | 3114 | 451294.96 | 6.9001 | 1.293 (1.241, 1.348) |  |
|  |  | ≥2 | 31845 | 2110 | 216545.34 | 9.7439 | 1.699 (1.619, 1.784) |  |
| Hypertension | No | 0 | 563484 | 15725 | 3925821.45 | 4.0055 | 1 (Ref.) | 0.8843 |
|  |  | 1 | 133744 | 5009 | 924637.62 | 5.4173 | 1.262 (1.222, 1.303) |  |
|  |  | ≥2 | 61356 | 3348 | 413979.36 | 8.0874 | 1.759 (1.694, 1.828) |  |
|  | Yes | 0 | 553260 | 24500 | 3836384.68 | 6.3862 | 1 (Ref.) |  |
|  |  | 1 | 164035 | 9983 | 1126047.31 | 8.8655 | 1.293 (1.263, 1.324) |  |
|  |  | ≥2 | 85002 | 7201 | 566673.76 | 12.7075 | 1.716 (1.670, 1.764) |  |
| Dyslipidemia | No | 0 | 677502 | 22313 | 4707721.42 | 4.7397 | 1 (Ref.) | 1 |
|  |  | 1 | 158259 | 7174 | 1088977.8 | 6.5878 | 1.282 (1.248, 1.317) |  |
|  |  | ≥2 | 69193 | 4474 | 462931.78 | 9.6645 | 1.733 (1.677, 1.791) |  |
|  | Yes | 0 | 439242 | 17912 | 3054484.72 | 5.8642 | 1 (Ref.) |  |
|  |  | 1 | 139520 | 7818 | 961707.13 | 8.1293 | 1.282 (1.248, 1.317) |  |
|  |  | ≥2 | 77165 | 6075 | 517721.33 | 11.7341 | 1.727 (1.676, 1.779) |  |
| CKD | No | 0 | 1047782 | 35337 | 7278172.44 | 4.8552 | 1 (Ref.) | 0.176 |
|  |  | 1 | 274662 | 12686 | 1892809.35 | 6.7022 | 1.280 (1.254, 1.307) |  |
|  |  | ≥2 | 132207 | 8701 | 888327.48 | 9.7948 | 1.752 (1.710, 1.795) |  |
|  | Yes | 0 | 68962 | 4888 | 484033.7 | 10.0985 | 1 (Ref.) |  |
|  |  | 1 | 23117 | 2306 | 157875.58 | 14.6064 | 1.292 (1.230, 1.358) |  |
|  |  | ≥2 | 14151 | 1848 | 92325.64 | 20.0161 | 1.627 (1.540, 1.718) |  |
| Insulin | No | 0 | 1051180 | 34545 | 7321430.26 | 4.7183 | 1 (Ref.) | 0.0878 |
|  |  | 1 | 269366 | 11943 | 1862956.16 | 6.4108 | 1.298 (1.271, 1.326) |  |
|  |  | ≥2 | 125410 | 7527 | 848787.57 | 8.8679 | 1.751 (1.707, 1.797) |  |
|  | Yes | 0 | 65564 | 5680 | 440775.88 | 12.8864 | 1 (Ref.) |  |
|  |  | 1 | 28413 | 3049 | 187728.77 | 16.2415 | 1.211 (1.159, 1.266) |  |
|  |  | ≥2 | 20948 | 3022 | 131865.54 | 22.9173 | 1.653 (1.580, 1.729) |  |
| OHA | <3 | 0 | 974781 | 32541 | 6758402.26 | 4.8149 | 1 (Ref.) | 0.0147 |
|  |  | 1 | 250015 | 11602 | 1717222.54 | 6.7563 | 1.290 (1.262, 1.318) |  |
|  |  | ≥2 | 120195 | 8085 | 804340.48 | 10.0517 | 1.767 (1.723, 1.812) |  |
|  | ≥3 | 0 | 141963 | 7684 | 1003803.88 | 7.6549 | 1 (Ref.) |  |
|  |  | 1 | 47764 | 3390 | 333462.39 | 10.1661 | 1.252 (1.203, 1.304) |  |
|  |  | ≥2 | 26163 | 2464 | 176312.63 | 13.9752 | 1.610 (1.538, 1.686) |  |
| DM Duration | <5 years | 0 | 838136 | 24593 | 5806917.03 | 4.2351 | 1 (Ref.) | <.0001 |
|  |  | 1 | 204891 | 8255 | 1406789.1 | 5.868 | 1.326 (1.293, 1.360) |  |
|  |  | ≥2 | 95146 | 5500 | 637922.35 | 8.6217 | 1.866 (1.811, 1.923) |  |
|  | ≥5 years | 0 | 278608 | 15632 | 1955289.1 | 7.9947 | 1 (Ref.) |  |
|  |  | 1 | 92888 | 6737 | 643895.83 | 10.4629 | 1.223 (1.188, 1.259) |  |
|  |  | ≥2 | 51212 | 5049 | 342730.77 | 14.7317 | 1.584 (1.533, 1.636) |  |

Competing risk model 5: Adjusted for age, sex, BMI, low income, smoking, drinking, regular exercise, hypertension, dyslipidemia, CKD, fasting glucose, duration of DM ≥5 years, insulin use, use of more than 3 types of oral hypoglycemic agents, and cardiovascular disease.

*Multiple testing was adjusted with Sidak method

Abbreviations: Q, quartile; IR, incidence rate; HR, hazard ratio; aHR, adjusted hazard ratio; CI, confidence interval; BMI, body mass index; DM, diabetes mellitus; CKD, chronic kidney disease; OHA, oral hypoglycemic agent.

**Supplemental Table S16. Subgroup analysis according to age (≥65 years)**

|  |  | Mental disorder | Age ≥65 years | | | | | |
| --- | --- | --- | --- | --- | --- | --- | --- | --- |
|  |  |  | N | Event | Duration | IR | Competing risk Model 5  HR (95% C.I) | *P for interaction |
| Sex | Male | 0 | 210939 | 24479 | 1346522.64 | 18.1794 | 1 (Ref.) | 0.1844 |
|  |  | 1 | 77849 | 10888 | 477411.13 | 22.8063 | 1.162 (1.136, 1.189) |  |
|  |  | ≥2 | 43522 | 7222 | 251747.44 | 28.6875 | 1.337 (1.301, 1.373) |  |
|  | Female | 0 | 160739 | 17063 | 1079669.79 | 15.8039 | 1 (Ref.) |  |
|  |  | 1 | 109382 | 13909 | 719769.48 | 19.3242 | 1.182 (1.156, 1.209) |  |
|  |  | ≥2 | 88690 | 13407 | 563413.56 | 23.796 | 1.404 (1.372, 1.436) |  |
| Income | Q2-4 | 0 | 296228 | 33198 | 1934852.23 | 17.1579 | 1 (Ref.) | 0.3813 |
|  |  | 1 | 151679 | 20048 | 972710.73 | 20.6104 | 1.167 (1.146, 1.187) |  |
|  |  | ≥2 | 107691 | 16679 | 666754.48 | 25.0152 | 1.360 (1.334, 1.386) |  |
|  | Q1 | 0 | 75450 | 8344 | 491340.2 | 16.9821 | 1 (Ref.) |  |
|  |  | 1 | 35552 | 4749 | 224469.89 | 21.1565 | 1.188 (1.147, 1.232) |  |
|  |  | ≥2 | 24521 | 3950 | 148406.52 | 26.6161 | 1.439 (1.385, 1.495) |  |
| Obesity | No | 0 | 212777 | 23637 | 1364383.03 | 17.3243 | 1 (Ref.) | 0.0007 |
|  |  | 1 | 106001 | 13723 | 663943.75 | 20.6689 | 1.144 (1.120, 1.169) |  |
|  |  | ≥2 | 76612 | 11590 | 461039.37 | 25.1389 | 1.332 (1.302, 1.363) |  |
|  | Yes | 0 | 158901 | 17905 | 1061809.4 | 16.8627 | 1 (Ref.) |  |
|  |  | 1 | 81230 | 11074 | 533236.87 | 20.7675 | 1.203 (1.175, 1.232) |  |
|  |  | ≥2 | 55600 | 9039 | 354121.62 | 25.5251 | 1.425 (1.389, 1.463) |  |
| Smoking | Non, Ex | 0 | 316484 | 34487 | 2084094.93 | 16.5477 | 1 (Ref.) | 0.9634 |
|  |  | 1 | 167420 | 21787 | 1078456.71 | 20.202 | 1.176 (1.156, 1.196) |  |
|  |  | ≥2 | 120945 | 18621 | 751119.47 | 24.791 | 1.382 (1.357, 1.408) |  |
|  | Current | 0 | 55194 | 7055 | 342097.5 | 20.6228 | 1 (Ref.) |  |
|  |  | 1 | 19811 | 3010 | 118723.91 | 25.3529 | 1.142 (1.094, 1.192) |  |
|  |  | ≥2 | 11267 | 2008 | 64041.53 | 31.3547 | 1.326 (1.261, 1.395) |  |
| Drinking | Non, Mild | 0 | 348429 | 38901 | 2277667.05 | 17.0793 | 1 (Ref.) | 1 |
|  |  | 1 | 179999 | 23797 | 1151973 | 20.6576 | 1.174 (1.155, 1.193) |  |
|  |  | ≥2 | 129140 | 20147 | 797064.93 | 25.2765 | 1.384 (1.360, 1.408) |  |
|  | Heavy | 0 | 23249 | 2641 | 148525.39 | 17.7815 | 1 (Ref.) |  |
|  |  | 1 | 7232 | 1000 | 45207.62 | 22.1202 | 1.199 (1.115, 1.289) |  |
|  |  | ≥2 | 3072 | 482 | 18096.07 | 26.6356 | 1.340 (1.216, 1.477) |  |
| Regular exercise | No | 0 | 287589 | 33329 | 1862486.56 | 17.8949 | 1 (Ref.) | 0.6343 |
|  |  | 1 | 150581 | 20568 | 954935.65 | 21.5386 | 1.168 (1.147, 1.188) |  |
|  |  | ≥2 | 109004 | 17393 | 665319.6 | 26.1423 | 1.364 (1.338, 1.389) |  |
|  | Yes | 0 | 84089 | 8213 | 563705.87 | 14.5697 | 1 (Ref.) |  |
|  |  | 1 | 36650 | 4229 | 242244.97 | 17.4575 | 1.183 (1.140, 1.228) |  |
|  |  | ≥2 | 23208 | 3236 | 149841.39 | 21.5962 | 1.432 (1.375, 1.492) |  |
| Hypertension | No | 0 | 105357 | 9677 | 694391.1 | 13.936 | 1 (Ref.) | 0.8509 |
|  |  | 1 | 48531 | 5219 | 314855.45 | 16.5759 | 1.160 (1.121, 1.200) |  |
|  |  | ≥2 | 32582 | 4218 | 204114.45 | 20.6649 | 1.408 (1.358, 1.460) |  |
|  | Yes | 0 | 266321 | 31865 | 1731801.34 | 18.3999 | 1 (Ref.) |  |
|  |  | 1 | 138700 | 19578 | 882325.17 | 22.1891 | 1.174 (1.153, 1.195) |  |
|  |  | ≥2 | 99630 | 16411 | 611046.54 | 26.8572 | 1.366 (1.340, 1.393) |  |
| Dyslipidemia | No | 0 | 222735 | 24700 | 1443166.3 | 17.1151 | 1 (Ref.) | 0.4814 |
|  |  | 1 | 100376 | 13097 | 634288.42 | 20.6483 | 1.158 (1.134, 1.183) |  |
|  |  | ≥2 | 64372 | 9833 | 389886.07 | 25.2202 | 1.349 (1.317, 1.382) |  |
|  | Yes | 0 | 148943 | 16842 | 983026.13 | 17.1328 | 1 (Ref.) |  |
|  |  | 1 | 86855 | 11700 | 562892.2 | 20.7855 | 1.188 (1.160, 1.216) |  |
|  |  | ≥2 | 67840 | 10796 | 425274.93 | 25.3859 | 1.404 (1.370, 1.439) |  |
| CKD | No | 0 | 299344 | 30653 | 1971915 | 15.5448 | 1 (Ref.) | <.0001 |
|  |  | 1 | 145290 | 17593 | 940717.15 | 18.7017 | 1.182 (1.160, 1.204) |  |
|  |  | ≥2 | 98714 | 14269 | 617228.67 | 23.1179 | 1.424 (1.395, 1.453) |  |
|  | Yes | 0 | 72334 | 10889 | 454277.44 | 23.9699 | 1 (Ref.) |  |
|  |  | 1 | 41941 | 7204 | 256463.47 | 28.0898 | 1.140 (1.106, 1.175) |  |
|  |  | ≥2 | 33498 | 6360 | 197932.33 | 32.1322 | 1.266 (1.227, 1.306) |  |
| Insulin | No | 0 | 337227 | 35740 | 2219481.42 | 16.1029 | 1 (Ref.) | 0.5364 |
|  |  | 1 | 164446 | 20464 | 1063004.15 | 19.2511 | 1.176 (1.156, 1.197) |  |
|  |  | ≥2 | 111498 | 16157 | 697402.68 | 23.1674 | 1.388 (1.362, 1.414) |  |
|  | Yes | 0 | 34451 | 5802 | 206711.01 | 28.0682 | 1 (Ref.) |  |
|  |  | 1 | 22785 | 4333 | 134176.47 | 32.2933 | 1.141 (1.097, 1.188) |  |
|  |  | ≥2 | 20714 | 4472 | 117758.32 | 37.9761 | 1.318 (1.267, 1.372) |  |
| OHA | <3 | 0 | 307361 | 32954 | 2006215.54 | 16.426 | 1 (Ref.) | 0.1221 |
|  |  | 1 | 152526 | 19385 | 974984.21 | 19.8824 | 1.173 (1.152, 1.194) |  |
|  |  | ≥2 | 107085 | 16177 | 660125.27 | 24.506 | 1.393 (1.366, 1.420) |  |
|  | ≥3 | 0 | 64317 | 8588 | 419976.89 | 20.4487 | 1 (Ref.) |  |
|  |  | 1 | 34705 | 5412 | 222196.41 | 24.3568 | 1.161 (1.122, 1.202) |  |
|  |  | ≥2 | 25127 | 4452 | 155035.73 | 28.716 | 1.310 (1.263, 1.359) |  |
| DM Duration | <5 years | 0 | 211729 | 21094 | 1384227.95 | 15.2388 | 1 (Ref.) | 0.0074 |
|  |  | 1 | 101744 | 12013 | 653074.99 | 18.3945 | 1.187 (1.160, 1.214) |  |
|  |  | ≥2 | 70089 | 9813 | 434551.66 | 22.5819 | 1.423 (1.388, 1.458) |  |
|  | ≥5 years | 0 | 159949 | 20448 | 1041964.48 | 19.6245 | 1 (Ref.) |  |
|  |  | 1 | 85487 | 12784 | 544105.62 | 23.4954 | 1.155 (1.129, 1.181) |  |
|  |  | ≥2 | 62123 | 10816 | 380609.34 | 28.4176 | 1.331 (1.300, 1.363) |  |

Competing risk model 5: Adjusted for age, sex, BMI, low income, smoking, drinking, regular exercise, hypertension, dyslipidemia, CKD, fasting glucose, duration of DM ≥5 years, insulin use, use of more than 3 types of oral hypoglycemic agents, and cardiovascular disease.

*Multiple testing was adjusted with Sidak method

Abbreviations: Q, quartile; HR, hazard ratio; aHR, adjusted hazard ratio; CI, confidence interval; BMI, body mass index; DM, diabetes mellitus; CKD, chronic kidney disease; OHA, oral hypoglycemic agent.
